# A Drug Repurposing Approach Reveals Targetable Epigenetic Pathways in *Plasmodium vivax* Hypnozoites

**DOI:** 10.1101/2023.01.31.526483

**Published:** 2023-06-19

**Authors:** S. P. Maher, M. A. Bakowski, A. Vantaux, E. L. Flannery, C. Andolina, M. Gupta, Y. Antonova-Koch, M. Argomaniz, M. Cabrera-Mora, B. Campo, A. T. Chao, A. K. Chatterjee, W. T. Cheng, C. A. Cooper, K. Cottier, M. R. Galinski, A. Harupa-Chung, H. Ji, S. B. Joseph, T. Lenz, S. Lonardi, J. Matheson, S. A. Mikolajczak, V. Padin-Irizarry, K. Pan, J. Peneau, J. Prudhomme, C. Roesch, A. A. Ruberto, S. S. Sabnis, C. L. Saney, J. Sattabongkot, S. Sereshki, S. Suriyakan, T. Moeller, R. Ubalee, Y. Wang, P. Wasisakun, J. Yin, C. W. McNamara, C. J. Joyner, F. Nosten, B. Witkowski, K. G. Le Roch, D. E. Kyle

**Affiliations:** 1 Center for Tropical & Emerging Global Disease, University of Georgia; Athens, GA, 30602, USA.; 2 Calibr, a division of The Scripps Research Institute; La Jolla, CA, 92037, USA.; 3 Malaria Molecular Epidemiology Unit, Institute Pasteur of Cambodia; Phnom Penh, 120 210, Cambodia.; 4 Novartis Institute for Tropical Diseases, Novartis Institutes for Biomedical Research; Emeryville, CA, 94608, USA.; 5 Shoklo Malaria Research Unit, Mahidol-Oxford Tropical Medicine Research Unit; Mae Sot, Tak, 63110, Thailand.; 6 Department of Molecular, Cell, and Systems Biology, University of California; Riverside, CA, 92521, USA.; 7 Center for Vaccines and Immunology, College of Veterinary Medicine, University of Georgia; Athens, GA, 30602, USA.; 8 International Center for Malaria Research, Education and Development, Emory Vaccine Center, Emory National Primate Research Center, Emory University; Atlanta, GA, 30329, USA.; 9 Medicines for Malaria Venture (MMV); Geneva, 1215, Switzerland.; 10 BioIVT Inc.; Westbury, NY, 11590, USA.; 11 Division of Infectious Diseases, Department of Medicine, Emory University; Atlanta, GA, 30329, USA.; 12 Department of Computer Science and Engineering, University of California; Riverside, CA, 92521, USA.; 13 Department of Microbiology and Immunology, University of Otago; Dunedin, 9016, New Zealand.; 14 Department of Biology, Clayton State University; Morrow, GA, 30260, USA.; 15 Mahidol Vivax Research Unit, Mahidol University; Bangkok, 10400, Thailand.; 16 Department of Entomology, Armed Forces Research Institute of Medical Sciences (AFRIMS); Bangkok, 10400, Thailand.; 17 Department of Chemistry, University of California; Riverside, CA, 92521; 18 Environmental Toxicology Graduate Program, University of California; Riverside, CA, 92521, USA.; 19 Centre for Tropical Medicine and Global Health, Nuffield Department of Medicine, University of Oxford; Oxford, OX3 7LG, UK.

## Abstract

Radical cure of *Plasmodium vivax* malaria must include elimination of quiescent ‘hypnozoite’ forms in the liver; however, the only FDA-approved treatments are contraindicated in many vulnerable populations. To identify new drugs and drug targets, we screened the Repurposing, Focused Rescue, and Accelerated Medchem library against *P. vivax* liver stages and identified the DNA methyltransferase inhibitors hydralazine and cadralazine as active against hypnozoites. We then used bisulfite sequencing and immunostaining to identify cytosine modifications in the infectious stage (sporozoites) and liver stages, respectively. A subsequent screen of epigenetic inhibitors revealed hypnozoites are broadly sensitive to histone acetyltransferase and methyltransferase inhibitors, indicating that several epigenetic mechanisms are likely modulating hypnozoite persistence. Our data present an avenue for the discovery and development of improved radical cure antimalarials.

## One-Sentence Summary

A drug repurposing screen reveals antihypertension drugs are active against *P. vivax* hypnozoites and epigenetic mechanisms play a role in hypnozoite quiescence.

## Introduction

Controlling malaria caused by *Plasmodium vivax* is complicated by the ability of *P. vivax* sporozoites, the infectious stage inoculated by mosquitoes, to invade hepatocytes and become quiescent (1). These quiescent ‘hypnozoites’ persist, undetectable, for months or even years before resuming growth and initiating a ‘relapse’ blood stage infection, leading to subsequent transmission back to mosquitoes (2). Hypnozoites are refractory to all antimalarials except the 8-aminoquinoline class, which cannot be administered to pregnant women or glucose-6-phosphate dehydrogenase-deficient individuals and are ineffective in persons with specific cytochrome P450 genotypes (3). New antimalarial drug discovery and development with a target chemo-profile for killing hypnozoites, termed radical cure, has only recently been made possible with the introduction of cell-based phenotypic screening platforms featuring a monolayer of hepatocytes infected with sporozoites, a portion of which go on to form hypnozoites (4). Protein target-based approaches for hypnozonticidal drug discovery are in their infancy as the transcriptome of hypnozoites has only recently been reported and robust methods for routine *in vitro* propagation and genetic manipulation of blood stages of *P. vivax* are still underdeveloped (5, 6).

To address the lack of radical cure drug leads and targets, we used our advanced *P. vivax* liver stage platform to screen the Repurposing, Focused Rescue, and Accelerated Medchem (ReFRAME) library (7). This library consists of approximately 12,000 developmental, approved, and discontinued drugs with the expectation that the repurposing of compounds with some optimization or regulatory success could expedite the decade-long path drugs typically progress through from discovery to licensure (8). To accomplish this screen, we assembled an international collaboration with laboratories in malaria-endemic countries whereby vivax-malaria patient blood was collected and fed to mosquitoes to produce sporozoites for infecting primary human hepatocytes (PHH) in screening assays performed on-site. As expected, few hits with anti-hypnozoite activity were detected since discovery of inhibitors for non-proliferating cells is notoriously difficult (9, 10). Interestingly, two structurally related compounds used for treating hypertension, hydralazine and cadralazine, were found effective at killing hypnozoites. As these inhibitors have been shown to modulate DNA methylation (11), we next pursued and confirmed the existence of cytosine modifications in *P. vivax* sporozoite and liver stages. To further investigate the role of epigenetics in hypnozoites, we screened an epigenetic inhibitor library using an improved version of the screening platform. Hypnozoites were found to be susceptible to several classes of epigenetic inhibitors, including six different histone deacetylase inhibitors and two inhibitors with targets regulating histone methylation. In lieu of traditional molecular biology methods for understanding this pathogen, our chemical biology approach reveals multiple, druggable pathways in *P. vivax* hypnozoites and sheds light on some of the processes underpinning their quiescence.

## Results

### ReFRAME library screening cascade, hit identification, and confirmation

**Fig. 1.**
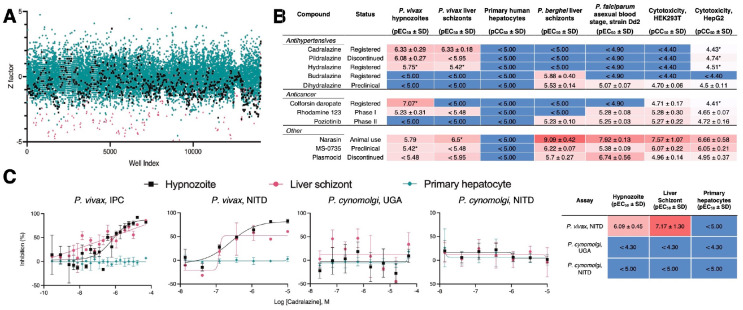
Hypnozonticidal hit detection and confirmation. (**A**) Index chart depicting the primary screen of the ReFRAME library against *P. vivax* hypnozoites in an 8-day assay. Hypnozoite counts were normalized by mean quantity per well for each plate (Z factor). Teal: library, black: DMSO, red: 1 μM monensin. (**B**) Primary screen hits were confirmed by dose-response in 8-day *P. vivax* liver stage assays and counterscreened against *P. berghei* liver schizonts, *P. falciparum* asexual blood stages (strain Dd2), HEK293T, and HepG2. Values represent pEC_50_ or pCC_50_ ± SD of all independent experiments (n=2–6) for which a pEC_50_ or pCC_50_ was obtained. (**C**) Dose-response curves for cadralazine against *P. vivax* and *P. cynomolgi* liver forms in 8-day assays at the IPC, UGA, and NITD. (B,C) Heat maps represent red as more potent and blue as inactive at highest dose tested. Asterisk (*) indicates only one independent experiment resulted in a calculated pEC_50_ or pCC_50_. (C) All replicate wells were plotted together from all independent experiments (n=3 for *P. vivax* at IPC, n=1 for *P. vivax* at NITD, n=2 for *P. cynomolgi* at UGA, and n=4 for *P. cynomolgi* at NITD), bars represent SEM.

Hypnozoites become insensitive to most antimalarials after five days in culture, indicating they mature following hepatocyte infection (12, 13). Importantly, development of hits with radical cure activity *in vivo* requires the screening against mature hypnozoites *in vitro* (14). While our 8-day *P. vivax* liver stage platform has been used for screening smaller libraries against mature hypnozoites (13), the size of the ReFRAME library presented a logistical challenge. We anticipated dozens of *P. vivax* cases, each with a unique genetic background, would be needed to produce the sporozoites required to screen the 40 microtiter plates containing the library, thus the library was split such that plates were screened at Shoklo Malaria Research Unit (SMRU) in Thailand and the Institute Pasteur of Cambodia (IPC). Ultimately, 36 *P. vivax* cases from either site were needed to complete the primary screen over the course of 18 months (Fig. 1A and fig. S1).

From our analysis of primary screen activity, we noted several hydrazinophthalazine vasodilators were potentially active (fig. S1). We then selected 72 compounds, including 10 hydrazinophthalazine analogs, for confirmation of activity and potency determination in a dose-response format, as well as counterscreening for additional antimalarial activity and cytotoxicity. Of the twelve hits which exhibited selective hypnozonticidal activity, cadralazine displayed the best combination of potency (pEC_50_ = 6.33 ± 0.33) and maximal inhibition near 100% (Fig. 1, B and C, and fig. S2). To ensure hits were not merely specific to our platform, select hits were communicated to the Novartis Institute for Tropical Diseases (NITD), where the activities of hydralazine and cadralazine were independently confirmed using a *P. vivax* case from southern Thailand and separate batches of compounds (Fig. 1C and fig. S3).

Currently, the gold-standard for *in vivo* anti-relapse efficacy is rhesus macaques infected with *Plasmodium cynomolgi*, a zoonotic, relapsing species closely related to *P. vivax* (15). To further pursue drug repurposing of hydrazinophthalazines, we sought to confirm and measure the potency of cadralazine and other ReFRAME hits against *P. cynomolgi* B strain hypnozoites *in vitro* using an 8-day assay featuring primary simian hepatocytes (PSH) at NITD. Surprisingly, hydralazine and cadralazine were found inactive when tested in three different PSH donors (Fig. 1C and fig. S3). This negative result was later confirmed in an 8-day, simianized version of the platform at the University of Georgia (UGA) using the *P. cynomolgi* Rossan strain infected into two different PSH lots (Fig. 1C). Furthermore, hydralazine and cadralazine were not identified as hits in any of the 112 screens of the ReFRAME published to date (ReFRAMEdb.org), suggesting these compounds are specific to the *P. vivax* liver stage assay.

### Immunofluorescent detection of DNA methylation in *P. vivax* and *P. cynomolgi* liver stages

**Fig. 2.**
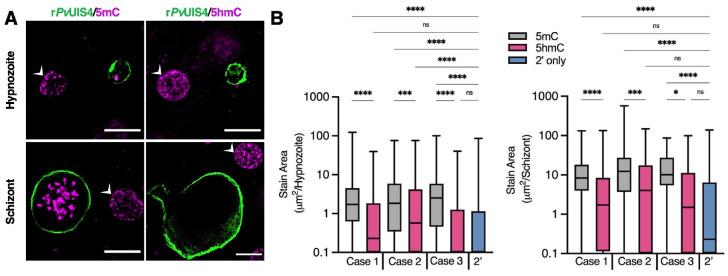
Cytosine modifications in *P. vivax* liver forms. (**A**) Immunofluorescent imaging of a 5mC-positive (left) or 5hmC-negative (right) *P. vivax* hypnozoite (top) and schizont (bottom) at day 6 post-infection. White arrows indicate hepatocyte nuclei positive for 5mC or 5hmC. Bars represent 10 μm. (**B**) High-content quantification of 5mC or 5hmC stain area within hypnozoites or schizonts from sporozoites generated from three different *P. vivax* cases. Significance determined using Kurskal-Wallis tests, for hypnozoites *H*(7) = 194.3, *p* <.0001, for schizonts *H*(7) = 88.66, *p* <.0001, with Dunn’s multiple comparisons, *p <.05, ***p <.001, ****p <.0001, ns = not significant. Line, box and whiskers represent median, upper and lower quartiles, and minimum-to-maximum values, respectively, of all hypnozoites (177 ≤ n ≤ 257) or all schizonts (30 ≤ n ≤ 142) in culture for each case, 2’ indicates a secondary stain only control.

Hydrazinophthalazines have been shown to involve direct inhibition of DNA methyltransferases (16) and hydralazine has also been used to study DNA methylation in the *Plasmodium falciparum* asexual blood stages (17). Investigations into blood-stage parasites methylation have identified the presence of low levels of 5-methylcytosine (5mC), 5-hydroxmethylcystosine (5hmC), and under-characterized 5hmC-like marks throughout the genome (17, 18). To confirm the possible mechanism of hydrazinophthalazines on hypnozoites, we first used immunofluorescence staining with commercial anti-5mC and anti-5hmC monoclonal antibodies to identify methylation patterns in *P. vivax* liver schizonts and hypnozoites at 6 days post-infection. We found clear evidence of 5mC, but not 5hmC, in both schizonts and hypnozoites, morphologically consistent with the presence of 5mC in the parasite’s nucleus (7) (Fig. 2A and figs. S4–S6). Due to expected 5mC and 5hmC signals from host hepatic nuclei, we opted to use high-content imaging (HCI) as an unbiased approach for quantifying 5mC signal within parasites. Image masks were generated to quantify the area of 5mC or 5hmC stain within each parasite (fig. S7), the values of which were then plotted as stain area per hypnozoite or per schizont (Fig. 2B). While some evidence of 5hmC-positive forms did appear from this analysis, the net area per parasite was found significantly lower than that quantified for 5mC-stained forms, indicating the relative level of 5mC marks predominate that of 5hmC.

Given the different susceptibility of *P. cynomolgi* hypnozoites to hydrazinophthalazines as compared to *P. vivax*, we performed HCI analysis of 5mC- and 5hmC-stained *P. cynomolgi* M/B-strain liver schizonts and hypnozoites at 8 and 12 days post-infection. Like *P. vivax*, we found both *P. cynomolgi* liver schizonts and hypnozoites are positive for 5mC, but not 5hmC. However, the 5mC stain morphology and intensity were relatively lower in *P. cynomolgi* hypnozoites versus *P. vivax* hypnozoites, indicating the possibility of different roles of methylation in the epigenetic network of these two species (fig. S8).

### Detection of cytosine modifications in *P. vivax* and *P. cynomolgi* sporozoites using liquid chromatography-tandem mass spectrometry and bisulfite sequencing

**Fig. 3.**
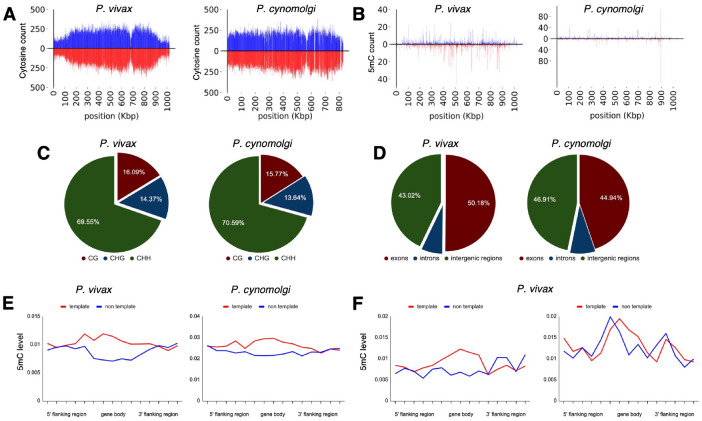
Density of cytosine and methylated cytosine (5mC) in sporozoites. (**A**) CG content of chromosome 1 for *P. vivax* and *P. cynomolgi*. The total number of cytosines was quantified on each strand using 1 kbp long non-overlapping windows. (**B**) The total number of methylated cytosines was quantified on each strand using 1 kbp long non-overlapping windows. (**C**) The number of 5mC present in all possible contexts (CG, CHG, and CHH) quantified throughout the genome of *P. vivax* and *P. cynomolgi*. (**D**) Repartitioned 5mC quantity within different compartments of the genome in *P. vivax* and *P. cynomolgi*. (**E**) Strand-specificity of 5mC for all genes in *P. vivax* and *P. cynomolgi*. Flanking regions and gene bodies were divided into five bins and the methylation level of each bin was averaged among all genes. Red: template strand, blue: non-template strand. (**F**) The previously reported mRNA abundance of *P. vivax* sporozoites was retrieved (20) and genes ranked. The 5mC levels in 5’ flanking regions, gene bodies, and 3’ flanking regions were placed into five bins and are shown for highly expressed (90th percentile, left) and weakly expressed (10th percentile, right) genes. Red: template strand, blue: non template strand.

We next sought to confirm the presence of cytosine methylation in the *P. vivax* and *P. cynomolgi* genomes using mass spectrometry and bisulfite sequencing. Initially, we assessed if these methodologies were plausible on *P. vivax* liver stages, but concluded that, without an available single live-cell approach, sequencing coverage of the parasite’s genome would be overwhelmed by the genomic material from the host cell and neighboring uninfected hepatocytes following the harvesting of liver stage cultures (19). We therefore collected sufficient genomic material from *P. vivax* and *P. cynomolgi* sporozoites to analyze the nucleoside mixture arising from the enzymatic digestion of genomic DNA by liquid chromatography-tandem mass spectrometry as well as for detection of DNA methyltransferase (DNMT) activity using a commercial *in vitro* DNA methylation assay (17). While we detected 5mC and DNMT activity in *Plasmodium*-enriched samples with these approaches, possible contamination by the mosquito’s microbiota could not be excluded (fig. S9). We next analyzed DNA methylation loci at single-nucleotide resolution using bisulfite sequencing of 3×10^7^ *P. vivax* sporozoites, generated from three different cases, as well as 3×10^7^ *P. cynomolgi* sporozoites (Fig. 3, A and B). A total of 161 and 147 million high-quality reads were sequenced for *P. vivax* and *P. cynomolgi* samples, respectively (fig. S9). The average 5mC level detected across all cytosines was 0.49% and 0.39% for *P. vivax* and *P. cynomolgi*, respectively. These percentages are comparable to the 0.58% methylation level detected in *P. falciparum* blood stages (17), but likely underestimate methylated loci considering the coverage we achieved (see methods).

We then monitored the distribution of detected 5mC along the *P. vivax* and *P. cynomolgi* chromosomes (figs. S10 and S11) and observed a stable methylation level throughout, including in telomeric and sub-telomeric regions. We further examined the context of genome-wide methylations and, similar to what we previously observed in *P. falciparum* (17), methylation was detected as asymmetrical, with CHH (where H can be any nucleotide but G) at 69.5% and 70.5%, CG at 16% and 15.7%, and CHG at 14.3% and 13.64%, for *P. vivax* and *P. cynomolgi*, respectively (Fig. 3C). We then measured the proportion of 5mC in the various compartments of gene bodies (exons, the introns, promoters, and terminators) as well as strand-specificity (Fig. 3, D and E). We observed a slightly increased distribution of 5mC in promoters and exons compared to the intronic region, as well as in the template versus non-template strand, in *P. vivax* and *P. cynomolgi*. These results were consistent with previous data obtained in *P. falciparum* and in plants (17). Such a strand specificity of DNA methylation patterns can affect the affinity of the RNA polymerase II and impact transcription. We therefore investigated the relationship between DNA methylation and transcription and examined the methylation level in the various compartments of gene bodies and compared it to the mRNA levels measured by RNA-seq in *P. vivax* sporozoites (20). We found a trend between methylation and mRNA abundance in the proximal promoter regions and the beginning of the gene bodies, with highly-expressed genes appearing hypomethylated and weakly-expressed genes hypermethylated (Fig. 3F). These results suggest that methylation level in proximal promoter regions as well as in the first exon of the genes may affect, at least partially, gene expression in malaria parasites. While these data will need to be further validated and linked to hypnozoite formation at a single-cell level, we have determined that 5mC is present at a low level in *P. vivax* and *P. cynomolgi* sporozoites and could control liver stage development and hypnozoite quiescence.

### Assay improvements and epigenetic inhibitor library screen

Upon completion of the ReFRAME and previously reported screens (13), we retroactively analyzed the screening platform’s performance and developed an improved protocol to address two shortcomings. First, we hypothesized that false negatives could arise when a true-positive compound results in nonviable forms which persist in culture and are counted during HCI; and that extending the assay endpoint by four days would allow more time for attenuated forms to be cleared from the culture (13). Second, because the ReFRAME primary screen was completed using two lots of PHH, we noted a few lot-specific results, such as differences in potency of the monensin control (fig. S12). We therefore added the phase I metabolism inhibitor 1-aminobenzotriazole to culture media on treatment days to minimize the effect of lot-specific hepatic metabolism on assay results (21) (fig. S13). Following development of the improved *in vitro* assay, this 12-day protocol was used to reconfirm twelve ReFRAME hits (fig. S14), resulting in confirmation of poziotinib--a primary screen hit that was previously confirmed in the NITD *P. vivax* assay only (Fig. 1B and fig. S3). Given the apparent importance of epigenetic mechanisms in hypnozoites, we obtained a commercially-available epigenetic inhibitor library and screened it against *P. vivax* liver stages at SMRU and IPC using the improved protocol (fig. S15). Confirmation of hits in dose-response assays resulted in selective potency for 11 epigenetic inhibitors targeting five different epigenetic mechanisms (Table 1).

## Discussion

Herein we demonstrate several significant advances that are prerequisites for radical cure antimalarial drug discovery and development, including the first report of screening a medium-sized compound library against mature hypnozoites as well as detection of novel hits with mechanisms unrelated to that of 8-aminoquinolines. Despite the success of the original screening platform protocol, the improvements described herein should be considered for other platforms grappling with the biological complexity of metabolically-active primary hepatocytes or using a phenotypic readout without a validated hypnozoite viability marker. This report also provides an opportunity to compare radical cure hits assayed against both *P. vivax* and *P. cynomolgi* hypnozoites. While the *P. cynomolgi*-infected rhesus macaque system has long been used as a model system for studying malaria relapse, the only class of compounds available to assess the predictive value of this model for chemotherapeutics are the 8-aminoquinolines, which lack a distinct parasite target (22–25). We found mixed results, with hydrazinophthalazines negative, and poziotinib positive, against *P. cynomolgi* hypnozoites. While further studies are needed to confirm if the target of any hit in this report is parasite- or host-directed, this would indicate there is sufficient diversity in gene expression, structural biology, or mechanisms of hepatic quiescence between *P. cynomolgi* and *P. vivax* hypnozoites that some hits will be species-specific. However, this result might also be attributed to differential metabolism in human and monkey hepatocytes (26). Regardless, these results highlight that *in vivo P. vivax* relapse models should be further developed and validated, as those hits lacking activity in *P. cynomolgi* might be abandoned for no other reason than the inability to demonstrate *in vivo* efficacy prior to first-in-human studies (27).

This report also adds to the broader discussion surrounding the successes and challenges of drug repurposing (28). Of the hits we identified, colforsin daropate, rhodamine 123, and poziotinib are used for cancer indications and, like the hydrazinophthalazines, have known human targets, implying that selectivity and off-target toxicity are hurdles to be addressed if repurposing for radical cure is to be successful. While direct repositioning of a known drug as a safe treatment for a new indication is the ideal outcome, it is also challenging. However, the identification of hits that can serve as advanced starting points for further optimization has potential for reducing the time and cost involved in developing an efficacious therapy. Alternatively, identification of tool compounds that delve into and reveal novel aspects of the biology of a disease can also promote further drug discovery approaches. As such, this report provides significant chemical biology leads into the essential nature of hypnozoite quiescence. Epigenetic control of pathogenic dormancy via DNA methylation has been described for cancer cells (29) and tuberculosis (30); and is a critical process in plants, which share evolutionary traits with *Plasmodium* (31). DNA methylation in the genus *Plasmodium* was first described in *P. falciparum* blood stages (17), and one type of modification has been demonstrated as positively associated with continuous gene expression (18). As technologies improve, it will be critical to validate the importance of such modifications in liver stages, including hypnozoites.

Several additional chemical biology leads were revealed in our screens. Five hydroxamic acid-containing inhibitors (panobinostat, AR42, abexinostat, givinostat and practinostat) and one natural product (raddeanin A) targeting histone deacetylase were found to selectively kill hypnozoites. Furthermore, the hit MI2, targeting the interaction between menin, a global regulator of gene expression, and MLL, a DNA-binding protein that methylates histone H3 lysine 4 (32), and the hit cyproheptadine, targeting SET-domain-containing lysine methyltransferase (33), highlight the interplay between DNA methylation and histone modifications (34) and are further evidence histone methylation regulates hypnozoites (20, 35). Other hits, including 666–15, targeting the transcription factor cAMP response element-binding protein (36), and the kinase inhibitors cerdulatinib and CCT241736, suggest transcription factors and kinase signaling may also play a role in establishing, maintaining, or exiting quiescence. Similarly, the cancer drug poziotinib inhibits HER2, a tyrosine protein kinase associated with the downregulation of apoptosis and metastasis (37). As we recently reported that host apoptotic pathways are downregulated in *P. vivax*-infected hepatocytes, poziotinib could act by increasing apoptotic pathways in infected host cells (19). The hit MS-0735, an analog of our previously reported hypnozonticidal hit MMV018983 (13), is a ribonucleotide-reductase (RNR) inhibitor used as an antiviral. Needed for producing deoxyribonucleosides for DNA synthesis, RNR is a peculiar mechanism for nonreplicating hypnozoites, however, it has been reported that RNR is critical for DNA damage repair (38) and is expressed in *P. vivax* liver schizonts and hypnozoites (19). Ongoing efforts to make available disruptive methods for studying *P. vivax*, including genetic manipulation and *in vivo* models for relapse, will enable further validation of these leads and a more comprehensive understanding of the mechanisms of hypnozoite quiescence.

## Supplementary Material

Supplement 1

Supplement 2

1

## Figures and Tables

**Table 1.** Confirmed hits from an epigenetic inhibitor library screened against *P. vivax* liver stages. Mean pEC_50_ or pCC_50_ and SD from two independent experiments. HDAC: histone deacetylase. CREB: cAMP response element-binding protein. FLT3: fms-like tyrosine kinase 3. SYK: spleen tyrosine kinase. JAK: Janus kinase. SETD: SET domain containing histone lysine methyltransferase.

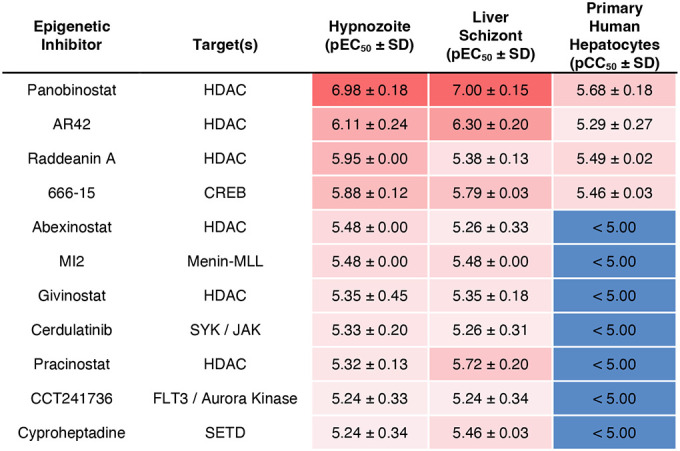

## Data Availability

For the purpose of Open Access, the authors have applied a CC BY public copyright license to any Author Accept Manuscript version arising from this submission. All bisulfite sequencing data generated in this study can be found in the Sequence Read Archive (SRA) at the NCBI National Library of Medicine (https://www.ncbi.nlm.nih.gov/sra) under the BioProject code PRJNA925570.
