# A Drug Repurposing Approach Reveals Targetable Epigenetic Pathways in *Plasmodium vivax* Hypnozoites

**DOI:** 10.1101/2023.01.31.526483

**Published:** 2024-03-25

**Authors:** S. P. Maher, M. A. Bakowski, A. Vantaux, E. L. Flannery, C. Andolina, M. Gupta, Y. Antonova-Koch, M. Argomaniz, M. Cabrera-Mora, B. Campo, A. T. Chao, A. K. Chatterjee, W. T. Cheng, E. Chuenchob, C. A. Cooper, K. Cottier, M. R. Galinski, A. Harupa-Chung, H. Ji, S. B. Joseph, T. Lenz, S. Lonardi, J. Matheson, S. A. Mikolajczak, T. Moeller, A. Orban, V. Padín-Irizarry, K. Pan, J. Péneau, J. Prudhomme, C. Roesch, A. A. Ruberto, S. S. Sabnis, C. L. Saney, J. Sattabongkot, S. Sereshki, S. Suriyakan, R. Ubalee, Y. Wang, P. Wasisakun, J. Yin, J. Popovici, C. W. McNamara, C. J. Joyner, F. Nosten, B. Witkowski, K. G. Le Roch, D. E. Kyle

**Affiliations:** 1 Center for Tropical & Emerging Global Disease, University of Georgia; Athens, GA, 30602, USA.; 2 Calibr, a division of The Scripps Research Institute; La Jolla, CA, 92037, USA.; 3 Malaria Molecular Epidemiology Unit, Institute Pasteur of Cambodia; Phnom Penh, 120 210, Cambodia.; 4 Novartis Institute for Tropical Diseases, Novartis Institutes for Biomedical Research; Emeryville, CA, 94608, USA.; 5 Shoklo Malaria Research Unit, Mahidol-Oxford Tropical Medicine Research Unit; Mae Sot, Tak, 63110, Thailand.; 6 Department of Molecular, Cell, and Systems Biology, University of California; Riverside, CA, 92521, USA.; 7 Center for Vaccines and Immunology, College of Veterinary Medicine, University of Georgia; Athens, GA, 30602, USA.; 8 International Center for Malaria Research, Education and Development, Emory Vaccine Center, Emory National Primate Research Center, Emory University; Atlanta, GA, 30329, USA.; 9 Medicines for Malaria Venture (MMV); Geneva, 1215, Switzerland.; 10 BioIVT Inc.; Westbury, NY, 11590, USA.; 11 Division of Infectious Diseases, Department of Medicine, Emory University; Atlanta, GA, 30329, USA.; 12 Department of Computer Science and Engineering, University of California; Riverside, CA, 92521, USA.; 13 Department of Microbiology and Immunology, University of Otago; Dunedin, 9016, New Zealand.; 14 School of Sciences, Clayton State University; Morrow, GA, 30260, USA.; 15 Mahidol Vivax Research Unit, Mahidol University; Bangkok, 10400, Thailand.; 16 Department of Entomology, Armed Forces Research Institute of Medical Sciences (AFRIMS); Bangkok, 10400, Thailand.; 17 Department of Chemistry, University of California; Riverside, CA, 92521; 18 Environmental Toxicology Graduate Program, University of California; Riverside, CA, 92521, USA.; 19 Centre for Tropical Medicine and Global Health, Nuffield Department of Medicine, University of Oxford; Oxford, OX3 7LG, UK.

## Abstract

Radical cure of *Plasmodium vivax* malaria must include elimination of quiescent ‘hypnozoite’ forms in the liver; however, the only FDA-approved treatments are contraindicated in many vulnerable populations. To identify new drugs and drug targets for hypnozoites, we screened the Repurposing, Focused Rescue, and Accelerated Medchem (ReFRAME) library and a collection of epigenetic inhibitors against *P. vivax* liver stages. From both libraries, we identified inhibitors targeting epigenetics pathways as selectively active against *P. vivax* and *P. cynomolgi* hypnozoites. These include DNA methyltransferase (DNMT) inhibitors as well as several inhibitors targeting histone post-translational modifications. Immunofluorescence staining of *Plasmodium* liver forms showed strong nuclear 5-methylcystosine signal, indicating liver stage parasite DNA is methylated. Using bisulfite sequencing, we mapped genomic DNA methylation in sporozoites, revealing DNA methylation signals in most coding genes. We also demonstrated that methylation level in proximal promoter regions as well as in the first exon of the genes may affect, at least partially, gene expression in *P. vivax*. The importance of selective inhibitors targeting epigenetic features on hypnozoites was validated using MMV019721, an acetyl-CoA synthetase inhibitor that affects histone acetylation and was previously reported as active against *P. falciparum* blood stages. In summary, our data indicate that several epigenetic mechanisms are likely modulating hypnozoite formation or persistence and provide an avenue for the discovery and development of improved radical cure antimalarials.

## One-Sentence Summary

Drug repurposing screens reveal several epigenetic inhibitors as active against *P. vivax* hypnozoites demonstrating that epigenetic pathways play a central role in hypnozoite quiescence.

## Introduction

Of the six species of *Plasmodium* that cause malaria in humans^1^, *Plasmodium vivax* is the most globally widespread^2^. Vivax malaria now accounts for the most malaria episodes in countries with successful falciparum malaria control programs^3^. Controlling vivax malaria is complicated by the ability of *P. vivax* sporozoites, the infectious stage inoculated by mosquitoes, to invade hepatocytes and become quiescent^4,5^. These quiescent ‘hypnozoites’ persist, undetectable, for months or even years before resuming growth and initiating a ‘relapse’ blood stage infection, leading to subsequent transmission back to mosquitoes^6^. New evidence suggests this transmission is expedited and silent as *P. vivax* liver merozoites can immediately form gametocytes instead of first having to establish an asexual stage blood infection, such as is the case for *P. falciparum*^7–10^. Clinically, a compound with radical cure efficacy is one that removes all parasites from the patient, including hypnozoites in the liver^11^.

Hypnozoites are refractory to all antimalarials except the 8-aminoquinolines, which were first identified over 70 years ago using low-throughput screening in avian malaria models^12^. Primaquine was the first 8-aminoquinoline widely-used for radical cure, however, efficacy is contingent on a large total dose administered in a 7–14 regimen, leading to adherence problems and infrequent use in malaria control programs of endemic countries^13^. Tafenoquine-chloroquine was developed from primaquine as an improved single-dose for radical cure^14^, but a recent clinical trial shows tafenoquine lacks efficacy when co-administered with the common antimalarial dihydroartemisinin-piperaquine, calling into question tafenoquine’s suitability in areas of high chloroquine resistance^15^. Furthermore, 8-aminoquinolines cannot be administered to pregnant women or glucose-6-phosphate dehydrogenase-deficient individuals and are ineffective in persons with specific cytochrome P450 genotypes^16^. For these reasons, the discovery and development of new chemical classes with radical cure activity are needed^17^.

Modern drug discovery typically relies on phenotypic screening and protein target identification^18^. For malaria, this approach ensures hits are acting on parasite targets and enables rational drug design, leading to several promising novel classes of antimalarials^19,20^. However, due to lower cost and higher feasibility, current high throughput screening for new antimalarials focuses almost entirely on blood or liver schizonts^21,22^. High throughput antimalarial screening with a target chemo-profile for killing hypnozoites has only recently been made possible with the introduction of cell-based phenotypic screening platforms featuring a monolayer of hepatocytes infected with sporozoites, a portion of which go on to form hypnozoites^23^. While the first hypnozonticidal hits from these platforms are just now being reported^24^, protein target identification approaches for hypnozonticidal drug discovery are in their infancy as the transcriptome of hypnozoites has only recently been reported and robust methods for genetic manipulation of *P. vivax* are still underdeveloped^25,26^.

To address the lack of radical cure drug leads and targets, we used our advanced *P. vivax* liver stage platform to first screen the Repurposing, Focused Rescue, and Accelerated Medchem (ReFRAME) library^7^. This library consists of approximately 12,000 developmental, approved, and discontinued drugs with the expectation that the repurposing of compounds with some optimization or regulatory success could expedite the decade-long path drugs typically progress through from discovery to licensure^27^. To accomplish this screen, we assembled an international collaboration with laboratories in malaria-endemic countries whereby vivax-malaria patient blood was collected and fed to mosquitoes to produce sporozoites for infecting primary human hepatocytes (PHH) in screening assays performed on-site. Interestingly, two structurally related compounds used for treating hypertension, hydralazine and cadralazine, were found effective at killing hypnozoites. Because these inhibitors have been shown to modulate DNA methylation^28,29^, we pursued and confirmed the existence of methyl-cytosine modifications in *P. vivax* sporozoite and liver stages. Having found in the ReFRAME screen a class of hits targeting an epigenetic pathway, we decided to confirm the importance of epigenetics in *P. vivax* hypnozoites and screened an additional commercial epigenetic inhibitor library using an improved version of our screening platform. Hypnozoites were found to be susceptible to several classes of epigenetic inhibitors, including six distinct histone deacetylase inhibitors and two inhibitors targeting histone methylation. To further assess the importance of histone acetylation in *P. vivax* liver stages, we tested inhibitors previously reported to be directly acting on *P. falciparum* acetyl-CoA synthetase, thereby modulating the pool of acetyl-CoA available for histone acetylation^30^. We found MMV019721 selectively kills *P. vivax* and *P. cynomolgi* hypnozoites, implicating acetyl-CoA synthetase is an additional hypnozonticidal drug target. This work demonstrates that in lieu of traditional molecular biology methods, our screening platforms identify multiple, druggable epigenetic pathways in hypnozoites and adds to the growing body of evidence that epigenetic features underpin biology in *P. vivax* and *P. cynomolgi* sporozoite and liver stages^25,31–33^.

## Results

### ReFRAME library screening cascade, hit identification, and confirmation

**Fig. 1.**
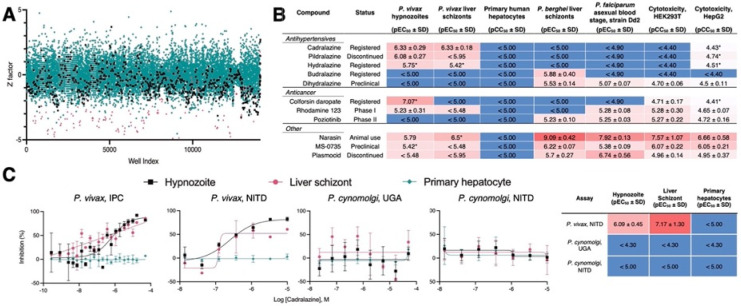
Hypnozonticidal hit detection and confirmation. (**A**) Index chart depicting the primary screen of the ReFRAME library against *P. vivax* hypnozoites in an 8-day assay. Hypnozoite counts were normalized by mean quantity per well for each plate (Z factor). Teal: library, black: DMSO, red: 1 μM monensin. (**B**) Primary screen hits were confirmed by dose-response in 8-day *P. vivax* liver stage assays and counterscreened against *P. berghei* liver schizonts, *P. falciparum* asexual blood stages (strain Dd2), HEK293T, and HepG2. Values represent pEC_50_ or pCC_50_ ± SD of all independent experiments (n=2–6) for which a pEC_50_ or pCC_50_ was obtained. (**C**) Dose-response curves for cadralazine against *P. vivax* and *P. cynomolgi* liver forms in 8-day assays at the IPC, UGA, and NITD. (B,C) Heat maps represent red as more potent and blue as inactive at highest dose tested. Asterisk (*) indicates only one independent experiment resulted in a calculated pEC_50_ or pCC_50_ (pEC_50_ is the inverse log of potency in M concentration, e.g. pEC_50_ 3 = 1 mM, pEC_50_ 6 = 1 μM, and pEC_50_ 9 = 1 nM). (C) All replicate wells were plotted together from all independent experiments (n=3 for *P. vivax* at IPC, n=1 for *P. vivax* at NITD, n=2 for *P. cynomolgi* at UGA, and n=4 for *P. cynomolgi* at NITD), bars represent SEM.

Chemical biology approaches have shown that hypnozoites become insensitive to most legacy antimalarials after 5 days in culture, indicating they must mature following hepatocyte infection^24,34^. Hypnozoite maturation was also noted in recent single-cell transcriptomic analyses of *P. vivax* liver stages, which demonstrate distinct population clusters of maturing and quiescent hypnozoites^10,25^. Importantly, discovery and development of hit compounds with radical cure activity *in vivo*, which includes elimination of hypnozoites in the liver of malaria patients^11^, requires screening against mature hypnozoites *in vitro*^35^. While our 8-day *P. vivax* liver stage platform, in which sporozoites are infected into primary human hepatocytes (PHH) and then allowed to mature for 5 days before being treated with test compound^36^, has been used for screening small libraries against mature hypnozoites^24^, the size of the ReFRAME library (12,823 compounds tested at 10 μM) presented a logistical challenge. We anticipated that dozens of *P. vivax* cases, each with a unique genetic background, would be needed to produce the sporozoites required to screen the 40 microtiter plates containing the library. To preclude the complex process of regular international shipments of infected mosquitoes, the *P. vivax* liver stage platform was successfully adapted and set up in research labs in two distinct malaria endemic areas, the Shoklo Malaria Research Unit (SMRU) in Thailand and the Institute Pasteur of Cambodia (IPC). The screening library was also divided between both sites to enable concurrent progress; ultimately 36 *P. vivax* cases from either site were needed to complete the primary screen over the course of 18 months (Figs. 1A, S1).

Some hits exhibited moderate selectivity and potency, with pEC_50_’s ranging from 5.42–7.07 (pEC_50_ is the inverse log of potency in M concentration, e.g. pEC_50_ 3 = 1 mM, pEC_50_ 6 = 1 μM, and pEC_50_ 9 = 1 nM) (Figs. 1B, S2). Colforsin daropate, rhodamine 123, and poziotinib are used to treat cancer and have known human targets, indicating that the targeted host pathways may be critical for hypnozoite persistence. As an example, poziotinib inhibits HER2, a tyrosine protein kinase associated with the downregulation of apoptosis and metastasis^37^. We recently reported that host apoptotic pathways are downregulated in *P. vivax*-infected hepatocytes^25^. Poziotinib could therefore act by upregulating apoptotic pathways in infected host cells. MS-0735, an analog of our previously reported hypnozonticidal hit, MMV018983^24^, is a ribonucleotide-reductase (RNR) inhibitor and used as an antiviral. The apparent need for nonreplicating hypnozoites to produce deoxyribonucleosides for DNA synthesis is peculiar. However, it has been reported that RNR is also critical for DNA damage repair^38^, is important for maintaining cancel cell dormancy^39^, and is expressed in *P. vivax* liver schizonts and hypnozoites^25^. We also rediscovered previously-reported hypnozonticidal compounds included in the library, including the ionophore narasin^24^ and the 8-aminoquinoline plasmocid^40^.

From our analysis of primary screen activity, we noted several hydrazinophthalazine vasodilators were potentially active (Fig. S1C). We selected 72 compounds for confirmation of activity against hypnozoites in a dose-response format, including 10 hydrazinophthalazine analogs. These compounds were counter-screened for additional antimalarial activity against *P. falciparum* blood stages and *P. berghei* liver schizonts. They were also tested for cytotoxicity against HEK293T and HepG2 human cell lines (Figs. 1B–C, S2). We confirmed that three hydrazinophthalazines analogs--cadralazine, pildralazine, and hydralazine--were active against mature hypnozoites, with cadralazine displaying the best combination of potency (pEC_50_ = 6.33 ± 0.33), maximal inhibition near 100%, and selectivity over PHH (> 21 fold), HEK293T (> 85 fold), and HepG2 (> 79 fold) cells (Figs. 1, B–C, S2A). Hydralazine, which was FDA-approved in 1953, is currently one of the world’s most-prescribed antihypertensives, and on the WHO list of essential medicines^41^. Cadralazine, which was developed in the 1980’s as an improvement over hydralazine, was abandoned due to side effects and only licensed in Italy and Japan^42^. Hydrazinophthalazines have been shown to inhibit human DNA methyltransferases (DNMT)^28,29^ and hydralazine has also been recently used to study potential DNA methylation patterns in the *P. falciparum* asexual blood stages^43^. Similar to our previous report^43^, these hydrazinophthalazines were inactive when tested against *P. berghei* liver schizonts and *P. falciparum* asexual blood stages, suggesting that hypnozoite quiescence may be biologically distinct from developing schizonts^24^. While hydrazinophthalazines may act on infected hepatocytes and not directly on the parasite, their distinct selectivity suggests that their effect is likely on a host or parasite pathways and not simply due to cytotoxicity in the host cell. Hydralazine and cadralazine were not identified as potential hits in any of the 112 bioassay screens of the ReFRAME published to date^44^, suggesting these compounds specifically target *P. vivax* liver stages and not promiscuously active compounds.

Methods for the robust culture of *P. vivax* hypnozoites were only recently reported, leading to several new reports on hypnozoite biology and radical cure drug discovery^7,45^. Consequentially, some hypnozoite-specific discoveries appear to be platform-specific^10,25^. Select hits were shared with the Novartis Institute for Tropical Diseases (NITD), where the activity and potency of cadralazine (pEC_50_ = 6.09 ± 0.45), hydralazine (pEC_50_ = 6.20), and poziotinib (pEC_50_ = 6.17) were independently confirmed in a similar 8-day *P. vivax* screening platform using a *P. vivax* case from southern Thailand. (Figs. 1C, S3A). Independent confirmation of these hits indicates their activities are not merely platform-specific and are, rather, more broadly descriptive of hypnozoite chemo-sensitivity.

Following our screening and hit confirmation, we investigated the potency, *in vivo* stability, and tolerability profile of our confirmed hits and chose cadralazine and hydralazine for repurposing as radical cure antimalarials. Currently, the gold-standard model for preclinical assessment of *in vivo* anti-relapse efficacy is rhesus macaques infected with *Plasmodium cynomolgi*, a zoonotic, relapsing species closely related to *P. vivax*^46^. Because we found cadralazine substantially more potent against hypnozoites than hydralazine, it was selected for a rhesus macaque pharmacokinetic study in which plasma levels were measured over 24 h following an oral dose of 1 mg/kg, which was calculated to be well-tolerated, and 30 mg/kg, which was calculated to likely cause drug-induced hypotension^47–49^. The 30 mg/kg dose resulted in maximum plasma concentration of 13.7 μg/mL (or 48.2 μM) and half-life of 2.19 ± 0.24 h, which was sufficient to cover the *in vitro* EC_90_ for several hours without noticeable side effects. (Fig. S4). As another prerequisite for *in vivo* validation, we next sought to confirm and measure the potency of cadralazine and other ReFRAME hits against *P. cynomolgi* B strain hypnozoites *in vitro* using an 8-day assay featuring primary simian hepatocytes (PSH) at NITD. While poziotinib was active against *P. cynomolgi* hypnozoites when tested two of three different PSH donor lots (pEC_50_ = 5.67 and 5.95), hydralazine and cadralazine were found inactive when tested in all three different PSH donor lots (Figs. 1C, S3B). This negative result was later confirmed in an 8-day, simianized version of the platform at the University of Georgia (UGA) using the *P. cynomolgi* Rossan strain infected into two different PSH lots (Fig. 1C). Altogether these data highlight potential difference between *P. vivax* and *P. cynomolgi* and challenge the gold-standard model for preclinical assessment of *in vivo* anti-relapse efficacy is rhesus macaques.

### Synergy between cadralazine and 5-azacytidine

**Fig. 2.**
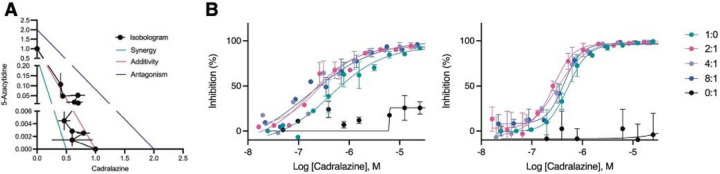
Synergistic effect of cadralazine and 5-azacytidine in *P. vivax* liver stage assays. (**A**) Isobologram of cadralazine and 5-azacytidine activity against hypnozoites in fixed ratios of 1:0, 8:1, 6:1, 4:1, 2:1, 1:1, 1:2, 1:4, 1:6, 1:8, and 0:1, bars represent SD of FICs from two independent experiments. (**B**) Dose-response curves for cadralazine at the most synergistic fixed ratios (2:1, 4:1, and 8:1) against hypnozoites. Cadralazine alone is represented as 1:0, 5-azacytidine alone is represented as 0:1 and plotted on the cadralazine chart for comparison. Left and right charts represent two independent experiments, bars represent replicate wells at each dose.

As molecular tools to validate drug target in *P. vivax* are limited, we further confirmed the possible mechanism of action of hydrazinophthalazines using drug combination studies to assess synergy, additivity or antagonsim^30^. We used 5-azacytidine, a known DNA methyltransferase inhibitor^50^, to investigate its effects on cadralazine treatment. When tested alone in dose-response from 50 μM, 5-azacytidine had no effect on hypnozoites. However, when added to cadralazine in fixed ratio combinations ranging from 8:1 to 1:8, 5-azacytdine increased the potency of cadralazine by ~2 fold across several combinations in two independent experiments (Figs. 2, S5). The most potent effect was detected using a 2:1 fixed ratio of cadralazine:5-azacytidine, resulting to an equivalent EC_50_ decrease from 470 nM to 216 nM.

### Immunofluorescent detection of DNA methylation in *P. vivax* and *P. cynomolgi* liver stages

**Fig. 3.**
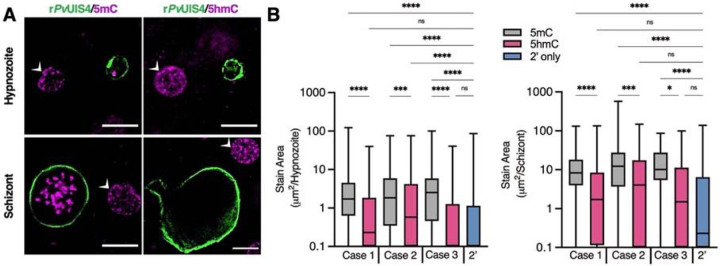
Cytosine modifications in *P. vivax* liver forms. (**A**) Immunofluorescent imaging of a 5mC-positive (left) or 5hmC-negative (right) *P. vivax* hypnozoite (top) and schizont (bottom) at day 6 post-infection. White arrows indicate hepatocyte nuclei positive for 5mC or 5hmC. Bars represent 10 μm. (**B**) High-content quantification of 5mC or 5hmC stain area within hypnozoites or schizonts from sporozoites generated from three different *P. vivax* cases. Significance determined using Kruskal-Wallis tests, for hypnozoites *H*(7) = 194.3, *p* <.0001, for schizonts *H*(7) = 88.66, *p* <.0001, with Dunn’s multiple comparisons, *p <.05, ***p <.001, ****p <.0001, ns = not significant. Line, box and whiskers represent median, upper and lower quartiles, and minimum-to-maximum values, respectively, of all hypnozoites (177 ≤ n ≤ 257) or all schizonts (30 ≤ n ≤ 142) in culture for each case, 2’ indicates a secondary stain only control.

**Fig. 4.**
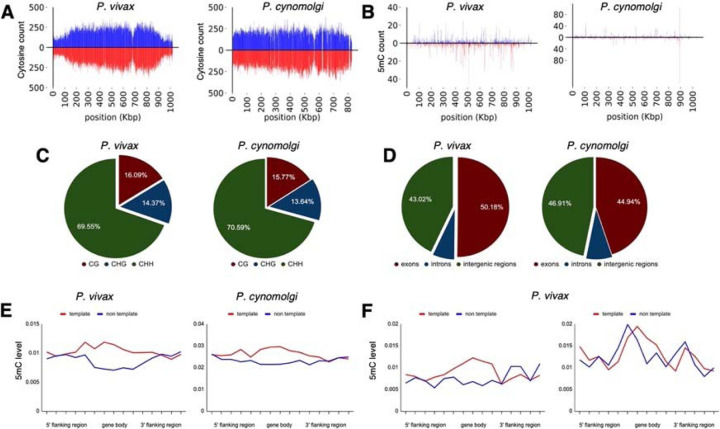
Density of cytosine and methylated cytosine (5mC) in sporozoites. (**A**) CG content of chromosome 1 for *P. vivax* and *P. cynomolgi*. The total number of cytosines was quantified on each strand using 1 kbp long non-overlapping windows. (**B**) The total number of methylated cytosines was quantified on each strand using 1 kbp long non-overlapping windows. (**C**) The number of 5mC present in all possible contexts (CG, CHG, and CHH) quantified throughout the genome of *P. vivax* and *P. cynomolgi*. (**D**) Repartitioned 5mC quantity within different compartments of the genome in *P. vivax* and *P. cynomolgi*. (**E**) Strand-specificity of 5mC for all genes in *P. vivax* and *P. cynomolgi*. Flanking regions and gene bodies were divided into five bins and the methylation level of each bin was averaged among all genes. Red: template strand, blue: non-template strand. (**F**) The previously reported mRNA abundance of *P. vivax* sporozoites was retrieved^22^ and genes ranked. The 5mC levels in 5’ flanking regions, gene bodies, and 3’ flanking regions were placed into five bins and are shown for highly expressed (90th percentile, left) and weakly expressed (10th percentile, right) genes. Red: template strand, blue: non template strand.

To further confirmed that cadralazine interacts with *P. vivax* target(s), we aimed to detect and quantify DNA methylation in the *P. vivax* and *P. cynomolgi* genomes. Previous studies had identified the presence of low level 5-methylcytosine (5mC), 5-hydroxmethylcystosine (5hmC), and 5hmC-like marks throughout the genome^43,51,52^. We first conducted an immunofluorescence staining assay using commercially available anti-5mC and anti-5hmC monoclonal antibodies to identify evidence of DNA methylation in *P. vivax* liver stages at 6 days post-infection. We found clear evidence of 5mC, but not 5hmC, in both schizonts and hypnozoites, morphologically consistent with the presence of 5mC in the parasite’s nucleus^7^ (Figs. 3A, S6–8). To segregate signals coming from the host hepatic nuclei, we used automated high content imaging analysis on hundreds of individual *P. vivax* liver stage parasites as an unbiased approach for quantifying 5mC signal within parasites. Image masks were generated to quantify the area of 5mC or 5hmC stain within each parasite (Fig. S9). The values were then plotted as stain area per hypnozoite or per schizont (Fig. 4B). While some evidence of 5hmC-positive forms did appear from this analysis, the net 5hmC area per parasite was found significantly lower when compared to 5mC signals (Kurskal-Wallis tests, for hypnozoites *H*(7) = 194.3, *p* <.0001, for schizonts *H*(7) = 88.66, *p* <.0001). Similar results on the ratio of 5hmC to 5mC were also recently reported in *P. falciparum* blood stages^53^, confirming that 5mC marks are the predominate DNA methylation marks in both species.

Given the different susceptibility of *P. cynomolgi* hypnozoites to hydrazinophthalazines as compared to *P. vivax*, we performed automated high content analysis of 5mC- and 5hmC-stained *P. cynomolgi* M/B-strain liver schizonts and hypnozoites at 8 and 12 days post-infection. Like *P. vivax*, we found both *P. cynomolgi* liver schizonts and hypnozoites are positive for 5mC, but not 5hmC. However, the 5mC stain morphology and intensity were relatively lower in *P. cynomolgi* hypnozoites versus *P. vivax* hypnozoites, suggesting potential divergence of DNA methylation pathways in these two species (Fig. S10).

### Detection of cytosine modifications in *P. vivax* and *P. cynomolgi* sporozoites using liquid chromatography-tandem mass spectrometry and bisulfite sequencing

We next sought to confirm the presence of cytosine methylation in the *P. vivax* and *P. cynomolgi* genomes using mass spectrometry and bisulfite sequencing. We initially assessed that without an available single cell sequencing approach, sequencing coverage of the parasite’s genome would be overwhelmed by the genomic material from the host cell as well as neighboring uninfected hepatocytes^25^. We therefore collected sufficient genomic material from *P. vivax* and *P. cynomolgi* sporozoites to analyze the nucleoside mixture arising from the enzymatic digestion of genomic DNA by liquid chromatography-tandem mass spectrometry as well as for detection of DNMT activity using a commercial *in vitro* DNA methylation assay^43^. While we detected 5mC and DNMT activity in *Plasmodium*-enriched samples with these approaches, possible contamination by the mosquito’s microbiota could not be excluded (Fig. S15A–B). We next analyzed DNA methylation loci at single-nucleotide resolution using bisulfite sequencing of 3×10^7^ *P. vivax* sporozoites, generated from three different cases, as well as 3×10^7^ *P. cynomolgi* sporozoites (Fig. 4, A–B). A total of 161 and 147 million high-quality reads were sequenced for *P. vivax* and *P. cynomolgi* samples, respectively (Fig. S11C). The average 5mC level detected across all cytosines was 0.49% and 0.39% for *P. vivax* and *P. cynomolgi*, respectively. These percentages are comparable to the 0.58% methylation level detected in *P. falciparum* blood stages^43^, but likely underestimate methylated loci considering the coverage we achieved (see methods).

We then monitored the distribution of detected 5mC along the *P. vivax* and *P. cynomolgi* chromosomes (Figs. S12–13) and observed a stable methylation level throughout the genomes, including in telomeric and sub-telomeric regions. We further examined the context of genome-wide methylations and, similar to what we previously observed in *P. falciparum*^43^, methylation was detected as asymmetrical, with CHH (where H can be any nucleotide but G) at 69.5% and 70.5%, CG at 16% and 15.7%, and CHG at 14.3% and 13.64%, for *P. vivax* and *P. cynomolgi*, respectively (Fig. 4C). We then measured the proportion of 5mC in the various compartments of gene bodies (exons, the introns, promoters, and terminators) as well as strand-specificity (Fig. 4, D–E). We observed a slightly increased distribution of 5mC in promoters and exons compared to the intronic region, as well as in the template versus non-template strand, in *P. vivax* and *P. cynomolgi*. These results were consistent with previous data obtained in *P. falciparum* and in plants^43,52^. Such a strand specificity of DNA methylation patterns can affect the affinity of the RNA polymerase II and impact transcription, thus we compared methylation levels to previously-report transcriptomic data from *P. vivax* sporozoites^32^. The 5mC levels in 5’ flanking regions, gene bodies, and 3’flanking regions were placed into five bins and compared to mRNA abundance, revealing an inverse relationship between methylation and mRNA abundance in the proximal promoter regions and the beginning of the gene bodies, with highly-expressed genes appearing hypomethylated and weakly-expressed genes hypermethylated (Fig. 4F). These results suggest that methylation level in proximal promoter regions as well as in the first exon of the genes may affect, at least partially, gene expression in malaria parasites. While these data will need to be further validated and linked to hypnozoite formation at a single-cell level, we have determined that 5mC is present at a low level in *P. vivax* and *P. cynomolgi* sporozoites and could control liver stage development and hypnozoite quiescence.

### Assay improvements and epigenetic inhibitor library screen

The success of the original screening platform protocol and secondary confirmation of several of our initial hits provided us an invaluable opportunity to develop an improved radical cure screening assay. The current iterations of our screening platform rely on high-content analysis of parasitophorous vacuole staining of the forms that persist up to the assay endpoint^7,54^. During the course of the ReFRAME primary screen we found the day 8 endpoint was sufficient for some hit compounds to act. However, other compounds like the 8-aminoquinolines exhibit a ‘delayed death’ phenotype, which leads to a false-negative result^24^. We therefore extended the assay by 4 days to allow attenuated forms to be cleared from the culture^24^. Also, as our screening assays were performed with multiple lots of PHH and PSH, we detected some lot-specific results, including small differences in activity of poziotinib and our monensin control (Figs. 1B, S3, S14A), possibly due to compound instability in the presence of hepatic metabolism. We therefore tested the metabolism inhibitor 1-aminobenzotriazole (1-ABT) in culture media to minimize the effect of lot-specific hepatic metabolism^55^. We used a cytochrome P450 functional assay specific to CYP3A4 and determined that 100 μM of 1-ABT was sufficient to completely reduce CYP3A4 activity in both basal and rifampicin-induced PHH (Fig. S15A–B). This effect was further confirmed and quantified by mass spectrometry after 1 h of treatment at 100 μM 1-ABT. We not only detected a 75% decrease in CYP3A4 activity, but also a more than 60% reduction of CYP2B6 and CYP2E1 activity along with lesser effects on CYP2C9, CYP1A2, and CYP2D6 (Fig. S15C). These changes were incorporated into our original 8-day protocol to design an improved 12-day assay^36^ that we then validated by re-testing 12 ReFRAME hits. The modified assay did not drastically affect the potency of most hits (Fig. S16A), but confirmed hypnozonticidal activity for poziotinib (pEC_50_ = 6.05), which had only been previously confirmed in *P. vivax* and *P. cynomolgi* assays performed at NITD only (Figs. 1B, S3, S16B). This assay was then use in all followup experiments.

To further confirm the importance of epigenetics in hypnozoites biology,^31^ we obtained a commercially-available library containing 773 compounds targeting various inhibitors of epigenetic enzymes or pathways. These compounds were tested at 10 μM against *P. vivax* liver stages at both SMRU and IPC sites (Fig. S17A). We confirmed our initial hits in dose-response assays resulting in selective hypnozonticidal potency for 11 compounds targeting five different epigenetic mechanisms (Tab. 1, Fig. S17B). This includes the histone deacetylase inhibitors panobinostat (pEC_50_ = 6.98 ± 0.18), AR42 (pEC_50_ = 6.11 ± 0.24), abexinostat (pEC_50_ = 5.48 ± 0.00), givinostat (pEC_50_ = 5.35 ± 0.45), practinostat (pEC_50_ = 5.32 ± 0.13), and raddeanin A (pEC_50_ = 5.95 ± 0.00). Histone methyltransferase inhibitor hits included MI2 (pEC_50_ = 5.48 ± 0.00), a compound that targets the interaction between menin (a global regulator of gene expression), and MLL (a DNA-binding protein that methylates histone H3 lysine 4^56^), and cyproheptadine (pEC_50_ = 5.24 ± 0.34), which targets the SET-domain-containing lysine methyltransferase^57^. These results corroborate our hypothesis that epigenetic pathways regulate hypnozoites^31,32^. Other hits, including 666–15 (pEC_50_ = 5.88 ± 0.12), an inhibitor of the transcription factor cAMP response element-binding protein^58^, and cerdulatinib (pEC_50_ = 5.33 ± 0.20), a kinase inhibitor, suggest that signaling pathways may also be important for quiescence^59^.

Having identified several histone deacetylase inhibitors as directly or indirectly active on hypnozoites, we screened compounds previously reported as inhibitors of *P. falciparum* acetyl-CoA synthetase (ACS), with downstream effects on histone acetylation^30^. We found that one compound, MMV019721, was selectively active on mature *P. vivax* hypnozoites (Tab. 1). Given the evidence MMV019721 is directly targeting *P. falciparum* ACS, this result suggests ACS also is a hypnozonticidal drug target. While the molecular techniques needed to confirm the direct interaction of MMV019721 and ACS in *P. vivax* are currently underdeveloped, our data supplement recent reports describing epigenetics as important regulators in *P. vivax and P. cynomolgi* at different stage of the parasite life cycle^25,32,33^.

## Discussion

Herein we demonstrate several significant advances that progress radical cure antimalarial drug discovery and development, including the first report of screening a medium-sized (>10,000) compound library against mature hypnozoites as well as detection of novel hits with mechanisms unrelated to that of 8-aminoquinolines. Identification of these hits was made possible following the establishment of a complex logistical operation in which the sporozoites used for screening were produced by feeding *P. vivax*-infected blood from malaria patient isolates to mosquito colonies at malaria research institutes in two countries in Southeast Asia. Our international collaboration overcame several logistical hurdles to obtain positive Z’ scores for most screening plates. Hits were also confirmed via dose-response, indicating that expanded screening directed against *P. vivax* liver stages is likely to produce more hypnozoite-specific hits (Figs. 1, 5, S1A–B, S17).

The only class of FDA-approved compounds for radical cure, the 8-aminoquinolines, were not discovered from *in vitro* drug screening. Instead, they were discovered using animal models, including the *P. cynomolgi*-infected rhesus macaque system^12^. The 8-aminoquionlines function through generation of reactive oxygen species affecting both the host and parasite, and lack a distinct parasite target^60–63^. As such, this work represents one of the first applications of a radical cure development pipeline to begin with *in vitro* screening against *P. vivax* hypnozoites and end with attempted confirmation using *P. cynomolgi* radical cure models. While our screen generated positive results against *P. vivax,* we found mixed results against *P. cynomolgi* hypnozoites *in vitro* (Figs. 1C, S3B). While further studies will be needed to confirm that target(s) of our hits are parasite- or host-directed and may influence parasite survival, our data show there is sufficient diversity in gene expression, structural biology, or mechanisms of hepatic quiescence between *P. cynomolgi* and *P. vivax* hypnozoites that some newly identified hits may be species-specific. While this result could also be attributed to differential metabolism in human and monkey hepatocytes^64^, the rhesus macaque radical cure model is currently considered as an important prerequisite for continued drug development, including efficacy testing in controlled human infections. The role of this model in the radical cure drug development cascade may need to be reevaluated as some compounds identified as promising for the radical cure of *P. vivax* may be abandoned too quickly due to the lack of activity against *P. cynomolgi*. This result highlights the need for further development and validation of *P. vivax*-specific animal models^65^. As a whole this report adds to the broader discussion surrounding the successes and challenges of drug repurposing^66^. While direct repositioning of a known drug as a safe treatment for a new indication is the ideal outcome, it can serve as advanced starting points for further optimization and has still the potential for reducing the time and cost involved in developing an efficacious therapy.

In addition to the identification of promising new hits and direction, we also confirmed that epigenetic control of pathogenic dormancy via DNA methylation is a pathway that could be potentially targeted by future antimalarials. This pathway has already been described for several disease agents capable of dormancy, including cancer cells^67^ and tuberculosis^68^. DNA methylation has also been validated as controlling critical processes in plants, which share evolutionary traits with *Plasmodium*^69^. DNA methylation in the genus *Plasmodium* was first described in *P. falciparum* blood stages^43^, and has been associated with gene expression, transcriptional elongation and parasite growth^51,52,70^. Previous experiment have shown that hydralazine can directly inhibits DNA methylation in nuclear extracts of blood stage parasites but also inhibit a recombinant functional fragment of the *P. falciparum* DNMT^43^. We pursued several biomolecular approaches to confirm that cadralazine may also interact with *P. vivax* DNMT in liver stage parasites. Due to technical limitations, we used a two-drug combination study in which the known DNMT inhibitor 5-azacytidine potentiated cadralazine against *P. vivax* hypnozoites (Figs. 2, S5). While we continue to develop new protocols and confirm the direct interaction of cadralazine with *P. vivax*, we successfully confirmed 5mC marks in *P. vivax* and *P. cynomolgi* liver stage parasites using both immunofluorescence and whole genome bisulfite sequencing assays (Figs. 3–4).

The current model of hypnozoite quiescence suggests RNA binding proteins (RBPs) drive hypnozoite formation by preventing translation of target mRNAs associated with schizogony^33^. In this model, histone acetylation results in euchromatin at the loci of RBPs, resulting in their expression and ongoing quiescence. Hypothetically, HDAC inhibitors would favor quiescence while a treatment that decreases histone acetylation would favor schizongony. This model somewhat contrasts with our present finding that HDAC inhibitors and the ACS inhibitor MMV019721 successfully kill hypnozoites *in vitro* (Tab. 1). It is, however, likely that the identified RBPs are part of broader gene networks which, when perturbed by sudden modulation of epigenetic feature such as DNA methylation and histone acetylation, results in a lethal level of dysregulation. While we still need to develop *P. vivax* transgenic lines to successful study hypnozoite biology and further validate potential drug targets^71,72^, the chemical probes that we described in this report could be used in combination with single-cell technology to more precisely perturb hypnozoites and refine our understanding of epigenetic pathways regulating hypnozoite formation and survival.

## Supplementary Material

Supplement 1

Supplement 2

Supplement 3

1

## Figures and Tables

**Table 1.** Additional epigenetic inhibitors with activity against *P. vivax* liver stages.

Epigenetic Inhibitor	Target(s)	Hypnozoite pEC_50_ ± SD	Liver Schizont pEC_50_ ± SD	PHH Nuclei pCC_50_ ± SD
Panobinostat	HDAC	6.98 ± 0.18	7.00 ± 0.15	5.68 ± 0.18
AR42	HDAC	6.11 ± 0.24	6.30 ± 0.20	5.29 ± 0.27
Raddeanin A	HDAC	5.95 ± 0.00	5.38 ± 0.13	5.49 ± 0.02
666–15	CREB	5.88 ± 0.12	5.79 ± 0.03	5.46 ± 0.03
Abexinostat	HDAC	5.48 ± 0.00	5.26 ± 0.33	< 5.00
MI2	Menin-MLL	5.48 ± 0.00	5.48 ± 0.00	< 5.00
Givinostat	HDAC	5.35 ± 0.45	5.35 ± 0.18	< 5.00
MMV019721	*P. falciparum* ACS	5.31 ± 0.03	5.25 ± 0.45	< 5.00
Cerdulatinib	SYK / JAK	5.33 ± 0.20	5.26 ± 0.31	< 5.00
Pracinostat	HDAC	5.32 ± 0.13	5.72 ± 0.20	< 5.00
CCT241736	FLT3 / Aurora Kinase	5.24 ± 0.33	5.24 ± 0.34	< 5.00
Cyproheptadine	SETD	5.24 ± 0.34	5.46 ± 0.03	< 5.00

HDAC: histone deacetylase. CREB: cAMP response element-binding protein. FLT3: fms-like tyrosine kinase 3. *P. falciparum* ACS: *P. falciparum* acetyl CoA synthetase. SYK: spleen tyrosine kinase. JAK: Janus kinase. SETD: SET domain containing histone lysine methyltransferase. Mean and standard deviation are from two or more independent experiments.

## Data Availability

For the purpose of Open Access, the authors have applied a CC BY public copyright license to any Author Accept Manuscript version arising from this submission. All bisulfite sequencing data generated in this study can be found in the Sequence Read Archive (SRA) at the NCBI National Library of Medicine (https://www.ncbi.nlm.nih.gov/sra) under the BioProject code PRJNA925570.
