# Supplementary material for: A Drug Repurposing Approach Reveals Targetable Epigenetic Pathways in Plasmodium vivax Hypnozoites
Source: bioRxiv. 2024 Mar 25:2023.01.31.526483. Originally published 2023 Jan 31. Preprint. [Version 3] doi: 10.1101/2023.01.31.526483 (PMC9915689; doi:10.1101/2023.01.31.526483)
Supplement: 1 [file NIHPP2023.01.31.526483V3-supplement-1.pdf]

bioRxiv preprint doi: https://doi.org/10.1101/2023.01.31.526483; this version posted March 25, 2024. The copyright holder for this preprint (which was not certified by peer review) is the author/funder, who has granted bioRxiv a license to display the preprint in perpetuity. It is made available under aCC-BY 4.0 International license.

Supplementary Materials for

A Drug Repurposing Approach Reveals Targetable Epigenetic Pathways in *Plasmodium vivax* Hypnozoites

Authors: S. P. Maher[1], M. A. Bakowski[2], A. Vantaux[3], E. L. Flannery[4], C. Andolina[5], M. Gupta[6], Y. Antonova-Koch[2], M. Argomaniz[7], M. Cabrera-Mora[8], B. Campo[9], A. T. Chao[4], A. K. Chatterjee[2], W. T. Cheng[7], E. Chuenchob[4], C. A. Cooper[1], K. Cottier[10], M. R. Galinski[8,11], A. Harupa-Chung[4], H. Ji[7], S. B. Joseph[2], T. Lenz[6], S. Lonardi[12], J. Matheson[13], S. A. Mikolajczak[4], T. Moeller[10], A. Orban[3], V. Padín-Irizarry[1,14], K. Pan[2], J. Péneau[3], J. Prudhomme[6], C. Roesch[3], A. A. Ruberto[1], S. S. Sabnis[7], C. L. Saney[7], J. Sattabongkot[15], S. Sereshki[12], S. Suriyakan[5], R. Ubalee[16], Y. Wang[17,18], P. Wasisakun[5], J. Yin[18], J. Popovici[3], C. W. McNamara[2], C. J. Joyner[7,8], F. Nosten[5,19], B. Witkowski[3], K. G. Le Roch[6], D. E. Kyle[1]*

*Corresponding author. Email: dennis.kyle@uga.edu

The PDF file includes:

- Materials and Methods
- Supplementary Text
- Figs. S1 to S18

Other Supplementary Materials for this manuscript include the following:

- Data S1
- Data S2
- Data S3

bioRxiv preprint doi: https://doi.org/10.1101/2023.01.31.526483; this version posted March 25, 2024. The copyright holder for this preprint (which was not certified by peer review) is the author/funder, who has granted bioRxiv a license to display the preprint in perpetuity. It is made available under aCC-BY 4.0 International license.

Materials and Methods

ReFRAME library description and plating

The ReFRAME library was curated by assembling a list of developmental and FDA-approved chemistry from three databases (GVK Excelra GoStar, Clarivate Integrity, and Citeline Pharmaprojects). The original library consisted of 36 384-well plates (ReF01-ReF36, Fig. S1A) containing 11,871 test compounds[27]. While the original library was being screened, an additional set of four 384-well plates (ReF38-ReF41, Fig. S1A) were added to the library, totaling 12,823 test compounds[44]. Source plates were made from the master library at Calibr at Scripps Research such that 3-5 µL of 10 mM solution was added to each well of a sterile, conical-bottom 384-well plate (Greiner Bio-One cat 784261). Most compounds were diluted in DMSO, however, a subset was diluted in water due to limited DMSO solubility. Plates were sealed and shipped on dry ice to SMRU and IPC and stored at -20 °C prior to use. Column 24 of each plate was filled with 5 µL DMSO to serve as negative control wells. Control compounds included 1 mM monensin (positive control for hypnozoite and schizont activity), 1 mM the phosphatidylinositol 4-kinase inhibitor (PI4Ki) KDU691 or MMV390048 (positive control for schizont activity), 1 mM atovaquone (negative control for radical cure activity) and 10 mM tafenoquine (clinically relevant control for hypnozoite activity)[7,24].

Ethical Approval for Human Subjects and Animal Use

The Thai human subjects protocols for this study were approved by the Institutional Ethics Committee of the Thai Ministry of Public Health and the Oxford Tropical Medicine Ethical Committee (TMEC 14-016 and OxTREC 40-14). The Cambodian human subjects protocols for this study were approved by the Cambodian National Ethics Committee for Health Research (100NECHR, 104NHECR, 111NECHR, 113NHECR and 237NHECR). Protocols conformed to the Helsinki Declaration on Ethical Principles for Medical Research Involving Human Subjects[73] and informed written consent was obtained for all volunteers or legal guardians. *P. cynomolgi* sporozoites were generated at Emory National Primate Research Center (ENPRC) using procedures approved by the Emory University Institutional Animal Care and Use Committee (PROTO201900110), as well as at UGA using procedures approved by UGA's Institutional Animal Care and Use Committee (A2020 03-002-Y3-A15). *P. cynomolgi* sporozoites were also produced at the Armed Forces Research Institute of Medical Science under an IACUC-approved animal use protocol in an AAALAC International-accredited facility with a Public Health Services Animal Welfare Assurance and in compliance with the Animal Welfare Act and other federal statutes and regulations relating to laboratory animals (22-10). *P. berghei* sporozoites were generated by the Sporocore at UGA using procedures approved by UGA's Institutional Animal Care and Use Committee (A2016 06–010–Y1–A0 and A2020 01-013-Y2-A3). Pharmacokinetic studies were conducted at WuXi AppTec Co., Ltd., in accordance with the WuXi IACUC standard animal procedures along with the IACUC guidelines that are in compliance with the Animal Welfare Act[74].

ReFRAME primary screen against *P. vivax* liver stages

bioRxiv preprint doi: https://doi.org/10.1101/2023.01.31.526483; this version posted March 25, 2024. The copyright holder for this preprint (which was not certified by peer review) is the author/funder, who has granted bioRxiv a license to display the preprint in perpetuity. It is made available under aCC-BY 4.0 International license.

The complete, step-by-step protocol for the *P. vivax* liver stage assay is published[36]. In summary, two days after assay plates (Greiner Bio-One cat 781956) were seeded with PHH, sporozoites were dissected from mosquito salivary glands and allowed to infect cultures. The ReFRAME library was screened using the original, 8-day radical cure assay, in which developing liver schizonts and mature, PI4Ki-insensitive hypnozoites were treated on days 5-7 post-infection[7,24]. On treatment days, a pintool was used to transfer 40 nL of compounds from the source plates to the assay plates. A single PHH lot, UBV, was first used for screening, however, once all available cryovials were used, screening was completed with lot BGW (Fig. S1A). Screening was initiated at SMRU until a second screening site was established at IPC, where all unfinished source plates were shipped and assayed. Some plates were assayed more than once in order to obtain a single run with a sufficient Z' factor of > 0.0 or two moderate-quality runs allowing for identification of reproducibly-active wells (Fig. S1B). Quantification of parasite growth was performed by fixing and staining cultures with recombinant mouse-anti *P. vivax* Upregulated in Infectious Sporozoites 4 (r*Pv*UIS4)[54]. followed by high-content imaging and analysis using an ImageXpress Micro (Molecular Devices) or Lionheart FX (Agilent). Hypnozoites were classified as forms of less than 125 μm^2 growth area.

<u>Normalization, hit selection, and dose-response confirmation in *P. vivax* liver stage assays</u>

Primary screening data were imported into Genedata Screener, Version 15.0.1-Standard and normalized to DMSO (neutral) and inhibitor (monensin) control-treated wells (neutral controls minus inhibitors). For four plates where the monensin control failed due to solubility issues combined with PHH lot variability (Fig. S14), data were normalized using the Robust Z-Score method, which calculates for each well the Robust Z-Score (number of standard deviations off the median) based on the statistics of the compound wells per plate. Genedata multiplicative pattern correction was applied to adjust for plate edge effects. Sixty-two most active (≥ 67% normalized inhibition of hypnozoite numbers) and non-toxic (≤ 40% host cell toxicity) compounds and 10 hydrazinophthalazines were selected for reconfirmation in an 8-point 1:3 dose response following the 8-day protocol with PHH lot BGW using a dose-response of monensin and nigericin as redundant positive controls. Once hydralazine and cadralazine were identified as reconfirmed hits, commercially available batches of powder were obtained (budralazine, Chemcruz cat sc-504334 batch D3019, cadralazine, Chemcruz cat sc-500641 batch B2417, and hydralazine, Selleckchem cat s2562 batch S256202) and used for additional reconfirmation runs using the same 8-day protocol (Fig. 1B-C, S2A).

<u>Hit confirmation in *P. cynomolgi* liver stage assays at UGA</u>

P. cynomolgi assays at UGA were performed using the step-by-step protocol for the *P. vivax* liver stage assay[36] with a few modifications. A Japanese macaque (*Macaca fuscata*) was intravenously infected with *P. cynomolgi* Rossan strain cryopreserved ring stage parasites[75] and allowed to reach patency. When parasitemia reached approximately 5,000 parasites per microliter, *An. dirus* mosquitoes were fed directly on the infected animal over a period of three to four days. The blood-fed mosquitoes were then checked for infection six to eight days by dissecting and staining midguts with 2% mercurochrome to detect oocysts. Two experiments were performed, one with PSH lot CWP, and one with PSH lot NPI. Two days after assay plates (Greiner Bio-One cat 781956) were seeded with 20,000 live PSH per well, sporozoites were

bioRxiv preprint doi: https://doi.org/10.1101/2023.01.31.526483; this version posted March 25, 2024. The copyright holder for this preprint (which was not certified by peer review) is the author/funder, who has granted bioRxiv a license to display the preprint in perpetuity. It is made available under aCC-BY 4.0 International license.

dissected from mosquito salivary glands at day 16 post-bloodmeal and allowed to infect cultures. Hits were confirmed using the same 8-day radical cure assay. On treatment days, a pin tool was used to transfer 40 nL of compounds from the source plates to the assay plates. Quantification of *P. cynomolgi* liver stage growth was performed by fixing and staining cultures with 100 ng/mL mouse monoclonal antibody 13.3 (anti-GAPDH) obtained from The European Malaria Reagent Repository (http://www.malariaresearch.eu) followed by high-content imaging and analysis using an ImageXpress Micro (Molecular Devices). Hypnozoites were classified as forms of less than 105 μm^2 growth area.

Hit confirmation in *P. cynomolgi* and *P. vivax* liver stage assays at NITD

Lots of both PSH and PHH were obtained from BioIVT. Hepatocytes were seeded at 22,000 cells per well in a 384-well plate (Corning cat 356667). Prior to and during the infection, the hepatocytes were cultured in BioIVT CP Medium (cat Z990003) with the addition of 1% PSN (Gibco cat 15640055) and 0.1 % gentamicin in the case of *P. vivax*. Two days post-seeding, the hepatocytes were infected with sporozoites dissected from the salivary glands of *An. dirus* mosquitoes. Sporozoites were collected in RPMI 1640 (KD Medical cat CUS-0645). Hepatocytes were infected with 10,000 sporozoites per well and spun for 5 minutes at 200 × *g*. Once the sporozoites were removed after 24 h of incubation, the culture media was exchanged to include 5% PSN in the case of *P. cynomolgi*. On days 4, 5, 6, and 7 post-infection, the hepatocytes received fresh compound addition in media. The cells were fixed on day 8 using 4% paraformaldehyde.

Liver stage parasites were detected by immunofluorescence assay. Hepatocytes were permeabilized for 1 h at room temperature in blocking buffer consisting of 2% Bovine Serum Albumin (Millipore Sigma cat A2153) and 0.2% Triton X-100 (Millipore Sigma cat 648466) in 1× PBS (Gibco cat 20012-027). For *P. cynomolgi* staining, the two in-house primary antibodies used were mouse anti-*Pc*UIS4 monoclonal at 10 ng/mL, and rabbit anti-*Pc*HSP70 polyclonal at 200 ng/mL. For *P. vivax* staining, rabbit anti-*Pv*MIF was used at 1:1000[76]. The primary antibodies were diluted in blocking buffer and incubated overnight at 4 °C. Hepatocytes were washed thrice with 1× PBS and then incubated with secondary antibodies (Invitrogen cat A11013, RRID: AB_2534080 and A11036, RRID: AB_10563566) used at a 1:1000 dilution and Hoechst 33342 (Invitrogen cat H3570) used at 2 µg/mL for 2 h at room temperature. After the incubation, the hepatocytes were washed 3 times with 1× PBS and were stored in 50 µL per well of 1× PBS prior to imaging on an ImageXpress Micro (Molecular Devices).

Confirmed hit counterscreens: *P. falciparum* asexual blood stage at Calibr

The SYBR Green I-based parasite proliferation assay[77] was used to determine the activity of compounds against the asexual blood stage of *P. falciparum* strain Dd2-HLH, a transgenic line expressing firefly luciferase[78]. Briefly, acoustic compound transfer (Labcyte Echo 550) was used to prepare assay ready plates to which parasites in assay medium were added and incubated with compounds for 72 h. SYBR Green I in lysis buffer was used as detection reagent. Fluorescence signal was read on the PHERAstar FSX plate reader (BMG Labtech). Compounds were tested in technical triplicates on different assay plates across three biological replicates performed on different days. Data were uploaded to Genedata Screener, Version 16.0.3-Standard and

bioRxiv preprint doi: https://doi.org/10.1101/2023.01.31.526483; this version posted March 25, 2024. The copyright holder for this preprint (which was not certified by peer review) is the author/funder, who has granted bioRxiv a license to display the preprint in perpetuity. It is made available under aCC-BY 4.0 International license.

normalized to DMSO (neutral) and inhibitor control-treated wells (neutral controls minus inhibitors), with 1.25 µM dihydroartemisinin used as a positive control. Dose curves (thirteen point, 1:3 dilution series) were fitted with the four parameter Hill Equation.

Confirmed hit counterscreens: *P. falciparum* asexual blood stage at UGA

Budralazine, cadralazine and hydralazine (same catalog and batches as above) were tested using the [^{3}H]-hypoxanthine drug susceptibility assay as previously described, with some modifications [79]. Strain W2[80,81] was grown in continuous culture using RPMI 1640 media containing 10% heat-inactivated type A+ human plasma, sodium bicarbonate (2.4 g/L), HEPES (5.94 g/L), and 4% washed human type A+ erythrocytes. Cultures were gassed with a 90% N2, 5% O2, and 5% CO2 mixture and incubated at 37 °C. Cultures were sorbitol synchronized to achieve >70% ring stage parasites[82]. Assay were started by establishing a 0.5−0.7% parasitemia and 1.5% hematocrit in complete media. Assays were performed in 96-well plates with a volume of 90 µL/well of parasitized erythrocytes and 10 µL/well of 10× test compound. Dihydroartemisinin was plated as a positive control and DMSO as a negative control. Assay plates were incubated in the above-mentioned gas mixture at 37 °C for 48 h; then, ^{3}H-hypoxanthine (185 MBq, Perkin Elmer cat NET177005MC) was added, and plates were incubated for another 24 h. After 72 h of incubation, the assay plates were frozen at −80 °C. Plates were allowed to thaw at room temperature before well contents were collected onto filtermats using a plate harvester (PerkinElmer). A Micro Beta liquid scintillation counter (PerkinElmer) was used to quantify radiation (counts-per-minute) representing relative parasite growth. Values were normalized to controls and plotted using CDD Vault. Potency values represent means of at least two independent experiments.

Confirmed hit counterscreens: *P. cynomolgi* asexual blood stage at UGA

Budralazine, cadralazine and hydralazine (same catalog and batches as above) were tested against *P. cynomolgi* DC strain using the [^{3}H]-hypoxanthine drug susceptibility assay as previously described, with some modifications[79]. *P. cynomolgi* was grown in continuous culture using RPMI 1640 +GlutaMAX media containing 20% heat-inactivated rhesus serum, hypoxanthine (32 mg/L), HEPES (7.15 g/L), additional glucose (2g/L), and 5% washed rhesus erythrocytes. Cultures were incubated at 37°C under mixed gas conditions of 90% N2, 5% O2, and 5% CO2. Schizonts were synchronized over a 60/20 Percoll gradient to achieve >90% late-stage parasites. Assays were started the following day when ring-stage parasites were present. Parasites were prepped for assay by establishing 0.5% ring-stage parasitemia and 2% hematocrit in complete media without hypoxanthine. Assays were performed in 96-well plates with a volume of 90 µL/well of parasitized erythrocytes and 10 µL/well of 10× test compounds. Compounds were plated from a starting concentration of 5 µM in an 11-point 1:2 dilution series and tested in duplicate. Uninfected RBCs were plated as a positive control and DMSO was used as a negative control. ^{3}H-hypoxanthine (185 MBq, Perkin Elmer cat NET177005MC) was then added to all wells and plates were incubated under the previously-mentioned conditions for 72 h. After 72 h the assay plates were frozen at -80 °C. Plates were thawed the following day at room temperature and well contents were collected onto filtermats using a plate harvester (PerkinElmer). A Micro Beta liquid scintillation counter (PerkinElmer) was used to quantify radiation (counts-per-minute) representing relative parasite growth. Values were normalized to

bioRxiv preprint doi: https://doi.org/10.1101/2023.01.31.526483; this version posted March 25, 2024. The copyright holder for this preprint (which was not certified by peer review) is the author/funder, who has granted bioRxiv a license to display the preprint in perpetuity. It is made available under aCC-BY 4.0 International license.

controls and plotted using CDD Vault. Potency values represent means of at least two independent experiments.

Confirmed hit counterscreens: *P. berghei* liver stage at Calibr

For *P. berghei* liver stage assays, a colony of *An. stephensi* mosquitoes was maintained in the UGA Sporocore using methods previously described[83]. In summary, adults were fed 5% dextrose (w/v) and 0.05% para-aminobenzoic acid (w/v) soaked into cotton pads and kept at a temperature of 27 °C, relative humidity of 75-85%, and a 12 h light/dark cycle. PbGFP-LUC_{CON} sporozoites were produced as previously described[83]. In summary, female C57BL/6 or Hsd:ICR(CD-1) mice (Envigo) were injected intraperitoneally with 5×10^6 - 5×10^7 blood stage parasites in 500 µL PBS 3-4 days before mosquito infections. Once parasitemia reached 2-6%, mice were anesthetized with 0.5 mL 1.25% 2,2,2-Tribromoethanol (v/v, Avertin, Sigma-Aldrich) and placed on top of cage of *An. stephensi* mosquitoes (3-7 days post-emergence) for 20 min to serve as an infectious bloodmeal. Infected mosquitoes were shipped to Calibr, where sporozoites were dissected out of mosquito salivary glands and used for luciferase-based infection assay as previously described[84]. Briefly, HepG2 cells (ATCC cat HB-8065, RRID: CVCL_0027) were infected with freshly dissected sporozoites. The infected cells were incubated with compounds of interest in 1536-well plates for 48 hours, and intracellular parasite growth was measured using bioluminescence. Compounds were tested in technical triplicates on different assay plates across three biological replicates performed on different days. Data were uploaded to Genedata Screener, Version 16.0.3-Standard and normalized to DMSO (neutral) and inhibitor control-treated wells (neutral controls minus inhibitors), with 1 µM KAF156 used as a positive control. Dose curves (thirteen point, 1:3 dilution series) were fitted with the four parameter Hill Equation.

Confirmed hit counterscreens: Mammalian cell cytotoxicity at Calibr

HepG2 (ATCC cat HB-8065, RRID: CVCL_0027) and HEK293T (ATCC cat CRL-3216, RRID: CVCL_0063) mammalian cell lines were maintained in Dulbecco's Modified Eagle Medium (DMEM, Gibco) with 10% heat-inactivated HyClone FBS (GE Healthcare Life Sciences), 100 IU penicillin, and 100 µg/mL streptomycin (Gibco) at 37 °C with 5% CO_2 in a humidified tissue culture incubator. To assay mammalian toxicity of hit compounds, 750 HepG2 and 375 HEK293T cells/well were seeded, respectively, in assay media (DMEM, 2 % FBS, 100 U/mL penicillin, and 100 µg/mL streptomycin) in 1536-well, white, tissue culture-treated, solid bottom plates (Corning cat 9006BC) that contained acoustically transferred compounds in a three-fold serial dilution starting at 40 µM. After a 72 h incubation, 2 µL of 50% Cell-Titer Glo (Promega cat G7573) diluted in water was added to the cells and luminescence measured on an EnVision Plate Reader (PerkinElmer).

Additional ReFRAME hit confirmation using an improved *P. vivax* liver stage assay

Twelve hits were re-confirmed using the 12-day radical cure assay, implementing three assay improvements[36]. First, 100 µM 1-ABT (Caymen Chem cat 15252) was added to media on treatment days to reduce hepatic metabolism. Second, the assay endpoint was extended 4 days to allow for nonviable liver stage forms to be cleared from cultures and therefore not be quantified during high-content imaging. Third, nigericin replaced monensin as the positive ionophore

bioRxiv preprint doi: https://doi.org/10.1101/2023.01.31.526483; this version posted March 25, 2024. The copyright holder for this preprint (which was not certified by peer review) is the author/funder, who has granted bioRxiv a license to display the preprint in perpetuity. It is made available under aCC-BY 4.0 International license.

control. Confirmation was performed with one independent experiment for all compounds except cadralazine, which was confirmed in four independent experiments.

Epigenetic inhibitor library screen against *P. vivax* liver stages

The Epigenetic Inhibitor library (Targetmol, cat L1200), containing 773 compounds at 10 mM, was purchased and re-plated in pintool-ready 384-well source plates with 200 µM nigericin and DMSO control wells. The library was screened using the 12-day radical cure assay noted above. The 24 hits exhibiting the highest inhibition against hypnozoites were replated in a dose-response for confirmation of activity in a 12-day radical cure assay as described above. Confirmation was performed with two independent experiments. The ACS inhibitors MMV019721 and MMV084978 were kindly provided by MMV and tested in dose-response in a 12-day radical cure assay as described above. Potency was determined from four independent experiments.

Assessment of effect of 1-ABT on hepatic cytochrome P450 3A4 activity

Two experiments were performed, one on uninduced PHHs, and another on rifampicin-induced PHHs (BioIVT, lot BGW). Cells were thawed and 18,000 live cells/well were seeded into collagen-coated 384-well plates as described above. Media was exchanged every-other-day until day 7 post-seed when media exchange included a dilution series of 1-ABT. One hour after addition of 1-ABT, cytochrome P450 3A4 activity (CYP3A4) was measured using a luciferin-IPA kit (Promega cat V9001) following the lytic protocol with 3 µM IPA. Lysed well contents were transferred to a white 384-well luminometer plate (Greiner Bio-One cat 201106) before reading on a Spectramax i3X (Molecular Devices) with a 1 sec integration time. In the second experiment, cells were similarly seeded and cultured before addition of 25 µM rifampicin (MP Biomedial cat BP2679-250), or an equivalent v/v DMSO vehicle control, in media on days 4 and 6. At day 7 post-seed, CYP3A4 activity was measured following addition of 1-ABT as above. The fold change was calculated between induced and uninduced wells at each 1-ABT dilution point.

Assessment of effect of 1-ABT on hepatic metabolism using mass spectrometry

Primary human hepatocytes (lot BGW, BioIVT) were thawed and 18,000 live cells/well were seeded into collagen-coated 384-well plates as described above. Media was exchanged every-other-day until day 7 post-seed when cells were treated with 100 µM 1-ABT, or an equivalent v/v vehicle control, in media for 1 h. Cells were then incubated with standard substrates for characterization of phase I and II hepatic metabolism, including: 30 µM 7-hydroxycoumarin (UGT/ST), 40 µM coumarin (CYP2A6), 500 µM chlorzoxazone (CYP2E1), 50 µM dextromethorphan (CYP2D6), 24 µM midazolam (CYP3A4/5), 500 µM S-mephenytoin (CYP2C19), 600 µM testosterone (CYP3A4), 1 mM tolbutamide (CYP2C9), 500 µM phenacetin (CYP1A2), or 400 µM bupropion (CYP2B6). The reaction was stopped at 1 h by addition of an equal volume of ice cold methanol. Metabolite formation was quantified using UPLC-MS/MS or LC-MS/MS (7-HC, 7-HCS, and 7-HCG). Samples were thawed, vortexed, and centrifuged for 5 min at 5000 rpm. Standards, controls, blanks, and study samples were added to an HPLC autosampler vial and injected into the UPLC-MS/MS or LC-MS/MS systems.

bioRxiv preprint doi: https://doi.org/10.1101/2023.01.31.526483; this version posted March 25, 2024. The copyright holder for this preprint (which was not certified by peer review) is the author/funder, who has granted bioRxiv a license to display the preprint in perpetuity. It is made available under aCC-BY 4.0 International license.

Analyses were run using an Acquity UPLC (Waters) or Agilent 1100 HPLC (Agilent) and Quattro premier XE (Waters) or Quattro Premier ZSpray (Waters) mass spectrometers. Quantification was performed using a quadratic least squares regression algorithm with $1/X^2$ weighting, based on the peak area ratio of substrate or metabolite to its internal standard. Metabolite formation rate was calculated as pmol/min/10^6 cells.

Combination drug studies in *P. vivax* liver stages

Powders of cadralazine (same batch as above), 5-azacytidine (Caymen Chem, cat 11164), and nigericin, were diluted to 50 mM, 50mM, and 200 µM, respectively, in DMSO, before being diluted to 100 µM, 100 µM, and 400 nM, respectively, in hepatocyte culture media (BioIVT, cat Z99029). Cadralazine and 5-azacytidine were then plated in the first column of two 96-well plates at volumetric ratios of 1:0, 8:1, 6:1, 4:1, 2:1, 1:1, 1:2, 1:4, 1:6, 1:8, and 0:1 such that the net volume per well was 200 µL (nigericin and DMSO controls were also diluted as such). Each mixture was then diluted in a 12-point, 2-fold dilution series by mixing 100 µL of mixture to 100 µL media in subsequent columns using a multichannel pipettor. A 384-well *P. vivax* liver stage assay plate was started using the 12-day protocol as above, and on day 5, 6, and 7 post-infection, media was removed from the 384-well plate using the inverted spin method[36] followed by addition of 20 µL of fresh media. Then, a multichannel pipettor was used to transfer 20 µL of the mixtures (made fresh daily) from the 96-well dilution series plates to the 384-well plates, thereby establishing a highest 1:0 and 0:1 treatment dose of 50 µM. The assay was fixed, stained, imaged, and parasite growth quantified as described above. Parasite growth data were normalized to the DMSO control and loaded into Prism (Graphpad) for curve fitting using the setting “log(inhibitor) vs. response -- variable slope (four parameters) least squares fit.” The EC_{50}s of each ratio were used to calculate Fractional Inhibitory Concentrations (FICs) and plot isobolograms as previously described[85].

Immunofluorescent staining of methyl-cytosine modifications in *P. vivax* liver stages

Sporozoites from three different *P. vivax* cases were infected into PHH lot BGW at day 2 post-seed (for case 1) or day 3 post-seed (for cases 2 and 3) in 384-well plates (Greiner Bio-One cat 781956) using the same methods for initiating *P. vivax* liver stage screening assays described above. Cultures were fixed at day 6 post-infection and stained with r*Pv*UIS4 and Hoechst 33342 as previously described[36,54]. Cultures were then stained for either with rabbit anti-5mC monoclonal antibody (clone RM231, ThermoFisher Scientific cat MA5-24694, RRID: AB_2665309) or rabbit anti-5hmC monoclonal antibody (clone RM236, ThermoFisher Scientific cat MA5-24695, RRID: AB_2665308) using methods adapted from those previously described[51]. In summary, cultures were re-permeablilized with 0.1% (v/v) Triton-X 100 for 20 min at room temperature and then washed thrice with 1× PBS. Chromatin was then denatured with 4 N HCl for 30 min at room temperature and washed thrice with 1× PBS. The denaturing reaction was then neutralized with 100 mM Tris (pH 8.0) for 10 min at room temperature and washed thrice with 1× PBS. Cultures were then quenched with 50 mM NH_4Cl for 10 min at room temperature and washed thrice with 1× PBS. Cultures were then blocked with 0.1% (v/v) Tween 20 and 2% (w/v) Bovine Serum Albumin for 10 min at room temperature and washed thrice with PBS. Cultures were then stained with either antibody diluted to 10 µg/mL in PBS overnight at 4 °C and washed thrice with 1× PBS. Cultures were then stained with 10 µg/mL Texas Red-

bioRxiv preprint doi: https://doi.org/10.1101/2023.01.31.526483; this version posted March 25, 2024. The copyright holder for this preprint (which was not certified by peer review) is the author/funder, who has granted bioRxiv a license to display the preprint in perpetuity. It is made available under aCC-BY 4.0 International license.

conjugated, goat anti-rabbit IgG secondary antibody (ThermoFisher Scientific, cat T-2767, RRID: AB_2556776) overnight at 4 °C and washed thrice with 1× PBS. For a negative stain control, a separate set of infected wells were prepared as above and stained with secondary antibody only (2' control, Fig. 3B). High resolution images of individual parasites and PHH nuclei were obtained by capturing 8 planes in the Z dimension using a 100× objective on Deltavision Core (GE Healthcare Life Sciences) and deconvoluted using softWoRx (GE Healthcare Life Sciences) (Fig. 3A, S6-8). An ImageXpress Micro high content imager was used to quantify methyl-cytosine modifications for the entire population of parasites from each case. A 20× objective was used to capture 25 fields of view from each well (covering the entire growth area) of the 384-well plate. Using the associated MetaXpress high content analysis software, the r*Pv*UIS4 stain from each parasite was used to define parasite objects, and the 5mC or 5hmC staining of host cell nuclei were used to define positive methyl-cytosine modification objects. The 2-dimensional area of intersection of both objects was then quantified for each parasite and forms less than 125 μm^2 were quantified as hypnozoites (Fig. 3B).

Immunofluorescent staining of methyl-cytosine modifications in *P. cynomolgi* liver stages

Japanese macaques (*Macaca fuscata*) were intravenously infected with *P. cynomolgi* M/B strain[86] and allowed to reach patency before skin feeding to *An. dirus* mosquitoes as described above. One round of macaque infection, mosquito dissection, and culture infection was performed with PSH lot NPI and a second round was performed with PSH lot NNF. Two days after assay plates (Greiner Bio-One cat 781956) were seeded with 20,000 primary simian hepatocytes per well, sporozoites were dissected from mosquito salivary glands at day 16 post-bloodmeal and allowed to infect cultures. Cultures were fixed on day 8 (experiment 1) or 12 (experiment 2) post-infection and stained for 5mC and 5hmC as described above. An ImageXpress Micro high content imager was used to quantify methyl-cytosine modifications for the entire population of *P. cynomolgi* liver stage parasites. A 20× objective was used to capture 25 fields of view from each well (covering the entire growth area) of the 384-well plate. Using the associated MetaXpress high content analysis software, the GAPDH stain from each liver stage parasite was used to define parasite objects, and the 5mC or 5hmC staining of host cell nuclei were used to define positive methyl-cytosine modification objects. The 2-dimensional area of intersection of both objects was then quantified for each parasite and forms less than 105 μm^2 were categorized as hypnozoites (Fig. S10).

Collection of *P. vivax* and *P. cynomolgi* sporozoites for methyl-cytosine characterization

For quantification of 5mC modification levels by mass spectrometry, sporozoites from 3 different *P. vivax* cases, numbering 18.7×10^6 from case 1, 10×10^6 from case 2, and 14.7×10^6 from case 3, were dissected from infected *An. dirus* mosquitoes at IPC as previously described[36] and cryopreserved as previously described[87]. For quantification of DMNT activity from nuclear extracts, sporozoites from 2 different *P. vivax* cases, numbering 21×10^6 from case 1 and 20×10^6 from case 2, were similarly dissected and cryopreserved. To serve as a negative control, salivary glands from uninfected mosquitoes at IPC were similarly dissected and cryopreserved. A total of 4.8×10^6 sporozoites for mass spec and 34.1×10^6 sporozoites for DNMT activity assays were also collected from *An. dirus* mosquitoes infected from feeding on a rhesus macaque infected with *P. cynomolgi* M/B strain at ENPRC and cryopreserved as described above. To serve as a

bioRxiv preprint doi: https://doi.org/10.1101/2023.01.31.526483; this version posted March 25, 2024. The copyright holder for this preprint (which was not certified by peer review) is the author/funder, who has granted bioRxiv a license to display the preprint in perpetuity. It is made available under aCC-BY 4.0 International license.

negative control, salivary glands and ovaries from uninfected mosquitoes at ENPRC were similarly dissected and cryopreserved. For mapping of methyl-cytosine modifications by bisulfite sequencing, sporozoites from three different *P. vivax* cases, numbering 9.8×10^6 from case 1, 12.3×10^6 from case 2, and 15.1×10^6 from case 3, were dissected from infected *An. dirus* mosquitoes at IPC and cryopreserved as described above. A total of 5.3×10^6 sporozoites were also collected from *An. dirus* mosquitoes infected from feeding on a rhesus macaque infected with *P. cynomolgi* M/B strain at ENPRC and cryopreserved as described above. Frozen sporozoites and salivary glands were shipped from IPC and ENPRC to University of California, Riverside on dry ice.

<u>Quantification of 5mC, 5hmC and 2'-deoxyguanosine (dG) in genomic DNA by LC-MS/MS/MS.</u>

Parasite pellets were lysed with 100 µl lysis buffer (20 mM Tris, pH 8.1, 20 mM EDTA, 400 mM NaCl, 1% SDS and 20 mg/ml proteinase K) and incubated at 55 ^{0}C overnight. Saturated solution of NaCl (0.5× volume of reaction mixture) was subsequently added to the digestion mixture and incubated at 55 °C for another 15 min. The samples were centrifuged at 14500 RCF for 30 min at 4 °C and the supernatant removed to a 1.5-mL microcentrifuge, genomic DNA (gDNA) was then precipitated with 2× volume of 100% chilled ethanol and resuspended in 95 µL water. Samples were then treated with 3 µL of 10 mg/mL RNase A and 2 µL of 25 units/µL RNase T1 and incubated overnight at 37 °C. gDNA was then extracted by chloroform/isoamyl alcohol solution, precipitated again with 100% chilled ethanol, and washed with 70% ethanol. The gDNA pellets were then dissolved in nuclease-free water. One µg of gDNA was enzymatically digested into mononucleosides using nuclease P1 and alkaline phosphatase. Enzymes in the digestion mixture were removed by chloroform extraction. The resulting aqueous layer was dried by using a SpeedVac and the dried residues were subsequently reconstituted in doubly distilled water. Approximately 5 ng of the DNA digestion mixture was injected for LC-MS/MS/MS analyses for quantifications of 5mC, 5hmC and dG. An LTQ XL linear ion-trap mass spectrometer equipped with a nano electrospray ionization source and coupled with an EASY-nLC II system (Thermo Fisher Scientific) was used for the LC-MS/MS/MS experiments. The amounts of 5mC, 5hmC and dG (in moles) in the nucleoside mixtures were calculated from area ratios of peaks found in the selected-ion chromatograms for the analytes over their corresponding isotope-labeled standards, the amounts of the labeled standards added (in moles), and the calibration curves. The final levels of 5mC and 5hmC, in terms of percentages of dG, were calculated by comparing the moles of 5mC and 5hmC relative to those of dG.

<u>Extraction of nuclear protein extracts</u>

Cryopreserved sporozoites, or parasites extracted from red blood cells by saponin lysis, were resuspended in 1 ml of cytoplasmic lysis buffer (20mM HEPES pH 7.9, 10 mM KCl, 1mM EDTA, 1mM EGTA, 1mM dithiothreitol (DTT), 0.5 mM AEBSF, 0.65% Igepal, 1× Roche complete protease inhibitor cocktail) and incubated for 10 min on ice. Nuclei were separated from cytoplasmic fraction by 10 min of centrifugation at 1500 RCF followed by two washes with cytoplasmic lysis buffer and one time wash with ice cold 1x PBS. Nuclei pellets were resuspended in 100 µl of nuclei lysis buffer (20 mM HEPES pH 7.9, 0.1 M NaCl, 1mM EDTA, 1 mM EGTA, 1mM DTT, 25 % glycerol, 0.5 mM AEBSF, 1× Roche complete protease inhibitor

bioRxiv preprint doi: https://doi.org/10.1101/2023.01.31.526483; this version posted March 25, 2024. The copyright holder for this preprint (which was not certified by peer review) is the author/funder, who has granted bioRxiv a license to display the preprint in perpetuity. It is made available under aCC-BY 4.0 International license.

cocktail) for 20 min at 4 °C with rotation. Nuclear extracts were cleared by 10 min of centrifugation at 6000 RCF. Protein concentration of nuclear extract was quantified by BCA assay and DNMT assays were performed immediately after estimation of protein concentration.

DNA methyltransferase assay

DNMT activity of nuclear extracts from *P. cynomolgi* sporozoites, *P. vivax* sporozoites, and uninfected mosquito salivary glands were measured using the Epiquik DNMT activity/inhibition assay ultra-kit (cat P-3010) following the manufacturer's instructions. Purified bacterial DNMT enzyme was used as a positive control. A blank control was used to subtract the residual background values. Each reaction was performed in duplicate. DNMT activity was measured in relative unit fluorescence per h per mg of protein for 10 min at 1 min intervals.

Bisulfite conversion and library preparation

P. cynomolgi and *P. vivax* sporozoites were lysed using 100 µl of lysis buffer containing 20mM Tris (pH 8.1), 20 mM EDTA, 400 mM NaCl, 1 % SDS (w/v) for 30 min at room temperature followed by addition of 20 µl of proteinase K (20 mg/ml) to the pellet and incubated at 55 °C overnight. The gDNA mixture was purified with phenol-chloroform followed by chloroform. Precipitation of gDNA was performed using chilled ethanol and treated with RNase A followed by another round of ethanol precipitation. 50 ng of unmethylated lambda DNA was added as a control to each sample before bisulfite conversion of the DNA. 500 ng of gDNA of each sample was used for the bisulfite conversion following the manufacturer's instructions (Epitect fast bisulfite conversion kit, Qiagen cat 59824). Libraries from bisulfite converted DNA were prepared using the Accel-NGS methyl-Seq DNA library kit (Swift biosciences cat 30024). Libraries were generated following the manufacturer's instructions and DNA was cleaned through SPRI select beads (Beckman Coulter). Libraries were sequenced using the NOVASeq platform.

DNA methylation analysis

Four sets of reads for *P. vivax* and *P. cynomolgi* were analyzed. Read qualities were checked with FastQC v0.11.8. FastQC indicated the presence of adapter contamination and overrepresented k-mers. As a result, (1) the first 9-14 base pairs were trimmed and (2) reads with overrepresented k-mers were discarded (see Fig. S11C for summary statistics after the cleaning step). Reads were mapped against the corresponding reference genomes downloaded from PlasmoDB (namely, PlasmoDB-48_Pfalciparum3D7, PlasmoDB-48_PcynomolgiB and PlasmoDB-48_PvivaxP01) using Bismark v0.22.2 with default parameters. To determine the bisulfite conversion rate, reads were also mapped against the lambda phage (see Fig. S11C for the conversion rate). Alignment files for the replicates were merged together using Samtools v1.9. Read methylation levels were obtained using Bismark v0.22.2 with default parameters (see Fig. S11C).

A cytosine in the genome was considered methylated if (1) the number of reads covering that cytosine was higher than a given threshold (10 for *P. falciparum*, 5 for *P. vivax* and 3 for *P. cynomolgi*) and (2) the ratio of methylated reads over all reads covering a cytosine was higher

bioRxiv preprint doi: https://doi.org/10.1101/2023.01.31.526483; this version posted March 25, 2024. The copyright holder for this preprint (which was not certified by peer review) is the author/funder, who has granted bioRxiv a license to display the preprint in perpetuity. It is made available under aCC-BY 4.0 International license.

than a given threshold (we chose 0.1 for this second threshold). Genome-wide cytosine density and methylated cytosine density in Fig. 4A-B were calculated in 1 kbp non-overlapping sliding windows using a custom script (available at https://github.com/salehsereshki/pyMalaria). The distribution of CG, CHG, CHH methylation in Fig. 4C-D were obtained by computing the number of methylated cytosines in each context over all the methylated cytosines. For the methylation analyses in genes in Fig. 4E, (1) 500 bp flanking regions and gene body were split into five bins, (2) methylation levels were averaged across all the genes using a custom script (available at the https://github.com/salehsereshki/pyMalaria). To study the correlation between cytosine methylation and gene expression, the same gene body computation was done for the 10% high and low expressed genes using a previously reported *P. vivax* transcriptome[32]. These plots are represented in Fig. 4F.

Supplementary Text

Immunofluorescent staining of 5mC and 5hmC in *P. vivax* blood stage parasites

An immunofluorescent staining approach has been used to detect both 5mC and 5hmC in *P. falciparum* blood stage parasites[51], thus we sought to confirm these marks in *P. vivax* blood stages. *P. vivax* blood samples were collected between 2017 and 2019 by active and passive case detection from individuals residing in Mondulkiri, Eastern Cambodia. The presence of *P. vivax* was determined using an RDT (CareStartTM Malaria Pf/pan RDTs, Accessbio) or microscopy and monoinfections were confirmed by RT-PCR using species-specific primers[88]. Venous blood used was collected in lithium heparin tubes and immediately processed on-site in a mobile laboratory. Leukocytes were depleted using NWF filters[89]. The leukocyte depleted parasitized red blood cells were cryopreserved using glycerolyte 57 solution (Baxter) and immediately stored in liquid nitrogen[90]. Blood isolates were thawed by addition of 12%, then 1.6%, and then 0.9% (w/v) NaCl solution followed by heparin treatment for 10 min at 37 °C. Blood stage parasites were then purified from thawed isolates using a KCl-Percoll density gradient[91] followed by a wash with RPMI and two washes with 1× PBS. Parasites were then fixed with 3% (v/v) paraformaldehyde and 0.01% (v/v) glutaraldehyde in 1× PBS for 1 h at 4 °C. After fixation, blood stage parasites were permeabilized, denatured, neutralized, quenched, and blocked as described above. Staining for 5mC and 5hmC was carried out as described above except the primary and secondary antibodies were diluted to 1 µg/mL instead of 10 µg/mL. Parasites were stained with 10 µg/mL Hoechst 33342 for 30 min at room temperature and then washed twice with 1× PBS after staining. Parasites were mounted on a coverslip and imaged with a 100× objective on a Leica DM250. While we did detect 5mC and 5hmC methylation in residual human white blood cells, we could not confirm positive 5mC or 5hmC staining in *P. vivax* blood stage parasites from these isolates (Fig. S18). These negative results could be due to one or more factors. First, while *P. falciparum* blood stage cultures can reach parasitemias above 10%, *P. vivax* blood stages cannot be propagated *in vitro* and the parasitemia of isolates is typically just above the level of detection. Second, *P. vivax* blood stage isolates were cryopreserved before staining and the stability of DNA methylation after cryopreservation is unknown. Third, the hydrochloric acid treatment needed to denature chromatin during the stain protocol causes red cells to aggregate, thereby making finding and imaging *P. vivax* blood stages difficult.

bioRxiv preprint doi: https://doi.org/10.1101/2023.01.31.526483; this version posted March 25, 2024. The copyright holder for this preprint (which was not certified by peer review) is the author/funder, who has granted bioRxiv a license to display the preprint in perpetuity. It is made available under aCC-BY 4.0 International license.

bioRxiv preprint doi: https://doi.org/10.1101/2023.01.31.526483; this version posted March 25, 2024. The copyright holder for this preprint (which was not certified by peer review) is the author/funder, who has granted bioRxiv a license to display the preprint in perpetuity. It is made available under aCC-BY 4.0 International license.

A

ReF#	Hyp/well	Sch/well	PvLS/well	Z' factor	Screen Location	PHH Lot	*P. vivax* Case
ReF01	111	77	188	N.A.	IPC	BGW	Pv847
ReF02	49	16	65	0.19	IPC	UBV	Pv595
ReF03	21	14	35	N.A.	SMRU	UBV	PID423955
ReF04	55	20	75	-0.04	IPC	UBV	Pv593
ReF05	87	61	148	0.17	IPC	UBV	Pv602
ReF06	27	33	60	0.11	SMRU	UBV	PID432054
ReF07	30	30	60	-0.02	SMRU	UBV	PID432054
ReF08	26	24	50	-0.03	SMRU	UBV	PID432054
ReF09	41	21	62	0.13	IPC	UBV	Pv602
ReF10	110	56	166	-0.01	IPC	BGW	Pv708
ReF11	24	21	45	0.06	SMRU	UBV	PID425583
ReF12	49	21	70	0.19	IPC	UBV	Pv602
ReF13	37	52	89	0.09	IPC	UBV	Pv603
ReF14	28	45	73	0.31	IPC	UBV	Pv603
ReF15	69	84	153	0.46	IPC	UBV	Pv603
ReF16	63	73	136	0.14	IPC	UBV	Pv603
ReF17	58	28	86	0.07	IPC	UBV	Pv606
ReF18	54	34	88	0.33	IPC	UBV	Pv606
ReF19	43	21	64	0.23	IPC	UBV	Pv606
ReF20	22	10	32	0.15	IPC	UBV	Pv608
ReF21	54	46	100	N.A.	IPC	BGW	Pv847
ReF22	76	55	131	0.04	IPC	UBV	Pv609
ReF23	40	40	80	0.06	SMRU	UBV	PID402389
ReF24	45	27	72	0.33	IPC	BGW	Pv849
ReF25	112	72	184	N.A.	IPC	UBV	Pv609
ReF26	117	66	183	N.A.	IPC	UBV	Pv609
ReF27	78	120	198	N.A.	IPC	UBV	Pv624
ReF28	107	61	168	0.38	IPC	BGW	Pv847
ReF29	69	54	123	0.32	IPC	BGW	Pv836
ReF30	65	36	101	N.A.	IPC	UBV	Pv623
ReF31	131	64	195	0.58	IPC	UBV	Pv611
ReF32	81	51	132	0.34	IPC	UBV	Pv609
ReF33	111	105	216	0.22	IPC	BGW	Pv708
ReF34	103	107	210	0.28	IPC	UBV	Pv624
ReF35	77	47	124	0.36	IPC	UBV	Pv609
ReF36	59	43	102	0.28	IPC	UBV	Pv609
ReF38	115	68	183	0.26	IPC	BGW	Pv836
ReF39	84	18	102	0.16	IPC	BGW	Pv838
ReF40	90	20	110	0.35	IPC	BGW	Pv846
ReF41	95	22	117	0.24	IPC	BGW	Pv846

B

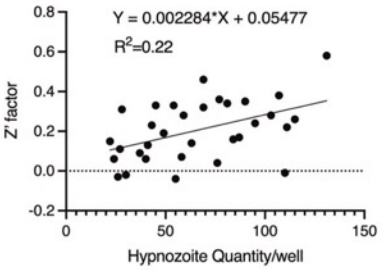

C

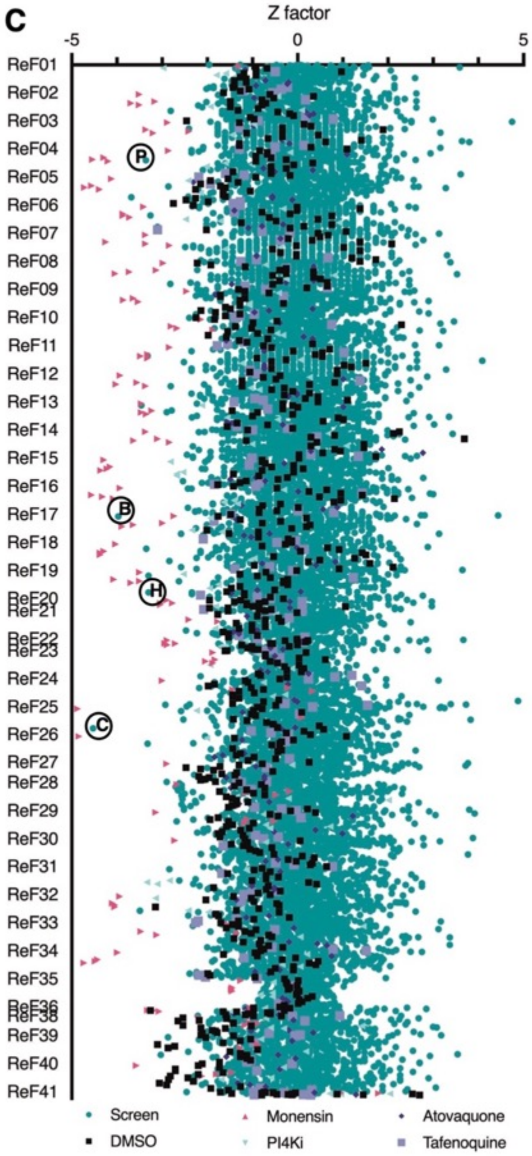

Fig. S1. ReFRAME screen run details. (**A**) Table of each ReFRAME plate (40 plates labelled 1-41, with 37 skipped) run metrics including average hypnozoites and schizont counts per well, Z' factor for 1 µM monensin wells, screening location (Shoklo Malaria Research Unit, Thailand, or Pasteur Institute of Cambodia) PHH lot used, and *P. vivax* patient isolate used. Due to an error during library plating, some plates contained only 1 well of monensin, preventing calculation of a Z' factor for those plates (listed as N.A.). (**B**) Simple linear regression correlating Z' factor with average hypnozoite count per well. (**C**) Index chart from Fig. 1A with phosphatidylinositol 4-kinase inhibitor (PI4Ki) KDU691 or MMV390048, tafenoquine, and atovaquone controls added. Teal circle: library, black square: DMSO, pink triangle: 1 µM monensin, light green inverted triangle: 1 µM P4Ki, black diamond: 1 µM atovaquone, purple square: 10 µM tafenoquine. Some hits discussed in this report are noted with black circles; P: poziotinib, B: budralazine, H: hydralazine, C: cadralazine.

bioRxiv preprint doi: https://doi.org/10.1101/2023.01.31.526483; this version posted March 25, 2024. The copyright holder for this preprint (which was not certified by peer review) is the author/funder, who has granted bioRxiv a license to display the preprint in perpetuity. It is made available under aCC-BY 4.0 International license.

A

Compound	Status	*P. vivax* hypnozoites ($pEC_{50} \pm SD$)	*P. vivax* liver schizonts ($pEC_{50} \pm SD$)	Primary human hepatocytes ($pCC_{50} \pm SD$)	*P. berghei* liver schizonts ($pEC_{50} \pm SD$)	*P. falciparum* asexual blood stage (Dd2) ($pEC_{50} \pm SD$)	*P. falciparum* asexual blood stage, strain W2 ($pEC_{50} \pm SD$)	*P. cynomolgi* asexual blood stage, strain DC ($pEC_{50} \pm SD$)	Cytotoxicity, HEK293T ($pCC_{50} \pm SD$)	Cytotoxicity, HepG2 ($pCC_{50} \pm SD$)
Antihypertensives										
Cadralazine	Registered	6.33 ± 0.29	6.33 ± 0.18	< 5.00	< 5.00	< 4.90	≤ 5.00	≤ 5.30	< 4.40	4.43*
Pildralazine	Discontinued	6.08 ± 0.27	≤ 5.95	< 5.00	< 5.00	< 4.90			< 4.40	4.74*
Hydralazine	Registered	5.75*	5.42*	< 5.00	< 5.00	< 4.90	≤ 5.00	≤ 5.30	< 4.40	4.51*
Budralazine	Registered	< 5.00	< 5.00	< 5.00	5.88 ± 0.4	< 4.90	≤ 5.00	≤ 5.30	< 4.40	< 4.40
Dihydralazine	Preclinical	< 5.00	< 5.00	< 5.00	5.53 ± 0.14	5.07 ± 0.07			4.7 ± 0.06	4.50 ± 0.11
Endralazine	Discontinued	< 5.00	< 5.00	< 5.00	< 5.00	< 4.90			4.51*	4.47*
Mopidralazine	Discontinued	< 5.00	< 5.00	< 5.00	< 5.00	< 4.90			< 4.40	< 4.40
Todralazine	Unknown	< 5.00	< 5.00	< 5.00	< 5.00	< 4.90			< 4.40	< 4.40
Dramedilol	Phase I	< 5.00	< 5.00	< 5.00	< 5.00	< 4.90			4.73 ± 0.06	4.60 ± 0.06
RGH-5526	Phase I	< 5.00	< 5.00	< 5.00	< 5.00	< 4.90			4.87 ± 0.19	4.67 ± 0.12
Anticancer										
Colforsin daropate	Registered	7.07*	< 5.00	< 5.00	< 5.00	< 4.90			4.71 ± 0.17	4.41*
Rhodamine 123	Phase I	5.23 ± 0.31	≤ 5.48	< 5.00	< 5.00	5.28 ± 0.08			5.28 ± 0.3	4.65 ± 0.07
PAN-811	Phase II	< 5.00	< 5.00	< 5.00	5.91 ± 0.29	5.66 ± 0.54			6.03 ± 0.23	5.77 ± 0.13
Poziotinib	Phase II	< 5.00	< 5.00	< 5.00	5.23 ± 0.1	5.25 ± 0.03			5.27 ± 0.22	4.72 ± 0.16
Other										
Narasin	Animal use	5.79 ± 0.2	6.50*	< 5.00	9.09 ± 0.42	7.92 ± 0.13			7.57 ± 1.07	6.66 ± 0.58
MS-0735	Preclinical	5.42*	≤ 5.48	< 5.00	6.22 ± 0.07	5.38 ± 0.09			6.07 ± 0.22	6.05 ± 0.21
Plasmocid	Discontinued	≤ 5.48	≤ 5.95	< 5.00	5.70 ± 0.27	6.74 ± 0.56			4.96 ± 0.14	4.95 ± 0.37
Controls	Assays									
KDU691	Blood, liver	< 5.52	7.08 ± 0.57	< 5.52	7.37 ± 0.16	7.46 ± 0.17			< 4.40	< 4.40
Dihydroartemisinin	Blood						7.42 ± 0.02			
Puromycin	Cytotoxicity				6.00 ± 0.1	7.12 ± 0.16			6.19 ± 0.18	6.05 ± 0.09
Monensin	*P. vivax* Liver	< 5.30	6.68 ± 0.13	< 5.30	9.77 ± 0.49	9.78 ± 0.49			8.00 ± 0.4	7.01 ± 0.63
Nigericin	*P. vivax* Liver	7.89 ± 0.15	8.38 ± 0.23	< 7.00	9.42 ± 0.71	8.74 ± 0.07			8.73 ± 0.34	7.67 ± 0.53

B

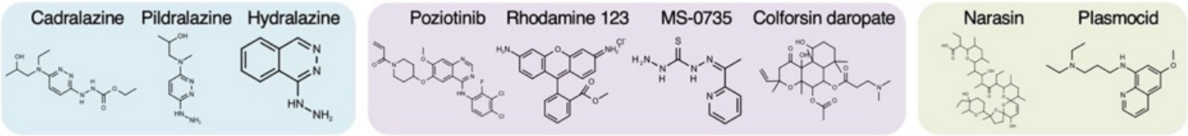

Fig. S2. Dose-response confirmation and counterscreens of primary screen hits and analogs. (**A**) Primary screen hits and structurally- or mechanistically-related compounds were tested by dose-response in 8-day *P. vivax* liver stage assays at Institute Pasteur of Cambodia and counterscreened against *P. berghei* liver schizonts, *P. falciparum* asexual blood stages, *P. cynomolgi* asexual blood stages, HEK293T, and HepG2. Values represent pEC_{50} or pCC_{50} ± SD of all independent experiments (n=2-6) for which a pEC_{50} or pCC_{50} was obtained. An asterisk (*) indicates only one independent experiment resulted in a calculated pEC_{50} or pCC_{50}. (**B**) Structures of hits which confirmed to be active against *P. vivax* hypnozoites in dose-response assays; blue: hydralazine analogs, purple: other novel hits, green: re-discovery of compounds previously demonstrated to have hypnozonticidal activity *in vitro* or antirelapse activity *in vivo.*

bioRxiv preprint doi: https://doi.org/10.1101/2023.01.31.526483; this version posted March 25, 2024. The copyright holder for this preprint (which was not certified by peer review) is the author/funder, who has granted bioRxiv a license to display the preprint in perpetuity. It is made available under aCC-BY 4.0 International license.

A

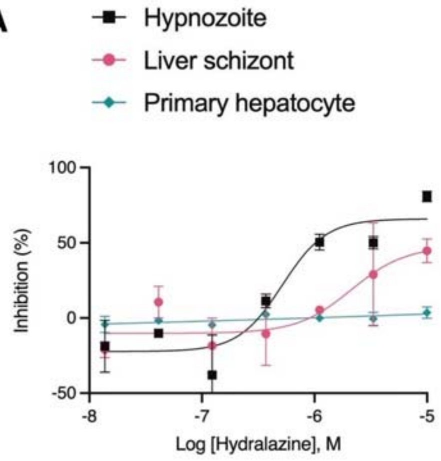

P. vivax assay, NITD	Hypnozoite (pEC_{50})	Liver Schizont (pEC_{50})	Primary human hepatocytes (pCC_{50})
Hydralazine	6.20	< 5.00	< 5.00
Poziotinib	6.17	5.74	< 5.00

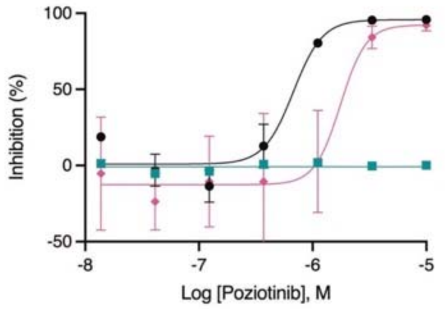

B

Compound	*P. cynomolgi* hypnozoite (pEC_{50})			*P. cynomolgi* schizont (pEC_{50})			Primary simian hepatocytes (pCC_{50})		
	NDO	NPI	XXJ	NDO	NPI	XXJ	NDO	NPI	XXJ
Cadralazine	< 5.00	< 5.00	< 5.00	< 5.00	< 5.00	< 5.00	< 5.00	< 5.00	< 5.00
Poziotinib	5.67	5.95	< 5.00	< 5.00	5.96	5.51	5.60	5.82	5.46
Hydralazine	< 5.00	< 5.00	< 5.00	< 5.00	< 5.00	< 5.00	< 5.00	< 5.00	< 5.00
Maduramicin	7.86	6.76	6.46	7.76	7.86	7.56	5.97	5.95	5.44

Fig. S3. Select ReFRAME hits confirmed at Novartis Institute for Tropical Diseases (NITD). (**A**) Dose-response curves for hydralazine and poziotinib against *P. vivax* liver forms assayed at NITD. All replicate wells were plotted together from a single independent experiment, bars represent SEM. (**B**) Potency data (pEC_{50}) for ReFRAME hits against *P. cynomolgi* liver forms assayed at NITD in primary simian hepatocyte (PSH) lots NDO, NPI, XXJ infected with one batch of *P. cynomolgi* sporozoites. Cytotoxicity (pCC_{50}) against PSH was measured using nuclei counts. Maduramicin is a positive control with activity against *P. cynomolgi* hypnozoites (A,B) Heat maps represent red as more potent and blue as inactive at highest dose tested.

bioRxiv preprint doi: https://doi.org/10.1101/2023.01.31.526483; this version posted March 25, 2024. The copyright holder for this preprint (which was not certified by peer review) is the author/funder, who has granted bioRxiv a license to display the preprint in perpetuity. It is made available under aCC-BY 4.0 International license.

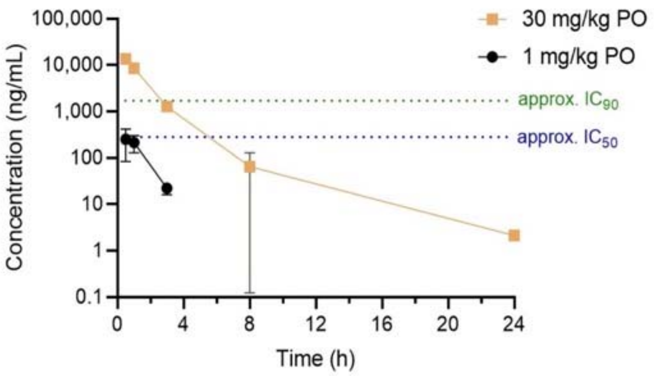

Fig. S4. Pharmacokinetics of cadralazine in nonhuman primates. Mean plasma concentration of cadralazine was measured in three males rhesus macaques after oral dosing. Plasma was collected following a 1 mg/kg dose, and again following a 30 mg/kg dose. Bars represent SD. The approximate IC_{50} and IC_{90} from *P. vivax* hypnozoite assays are indicated.

bioRxiv preprint doi: https://doi.org/10.1101/2023.01.31.526483; this version posted March 25, 2024. The copyright holder for this preprint (which was not certified by peer review) is the author/funder, who has granted bioRxiv a license to display the preprint in perpetuity. It is made available under aCC-BY 4.0 International license.

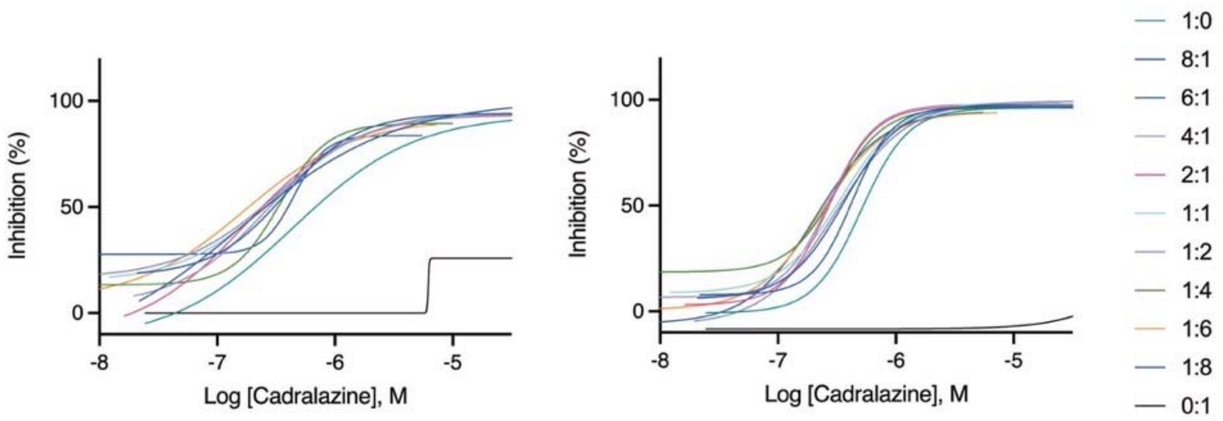

Fig. S5. Synergistic effect of cadralazine and 5-azacytidine in *P. vivax* liver stage assays. Dose-response curves for cadralazine with all fixed ratios of 5-azacytidine against *P. vivax* hypnozoites. Cadralazine alone is represented as 1:0, 5-azacytidine alone is represented as 0:1 and plotted on the cadralazine chart for comparison. Left and right charts represent two independent experiments.

bioRxiv preprint doi: https://doi.org/10.1101/2023.01.31.526483; this version posted March 25, 2024. The copyright holder for this preprint (which was not certified by peer review) is the author/funder, who has granted bioRxiv a license to display the preprint in perpetuity. It is made available under aCC-BY 4.0 International license.

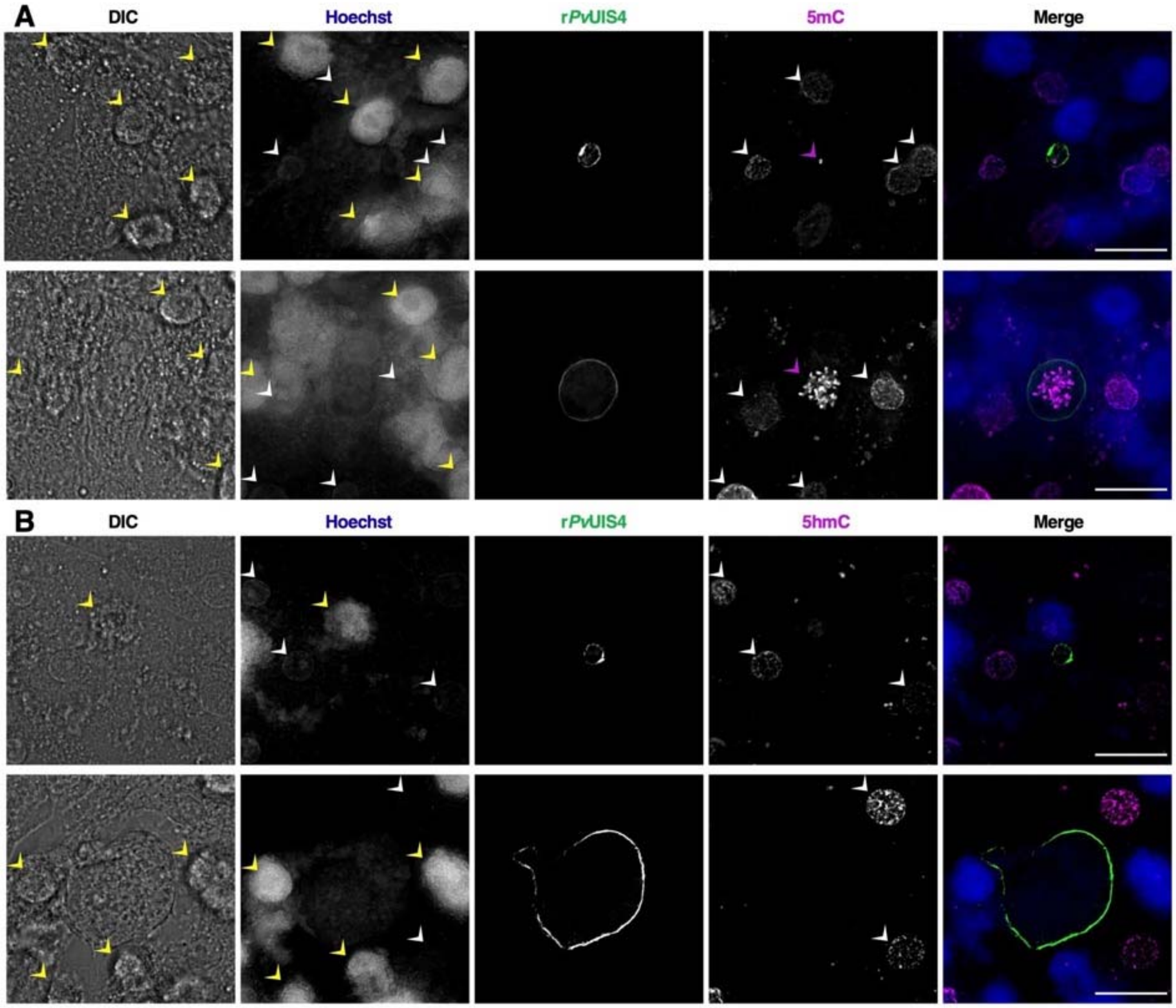

Fig. S6. Cytosine modifications in *P. vivax* liver forms, full panels from Case 1 (also shown in Fig. 3). (**A**) Immunofluorescent imaging of a 5mC-positive *P. vivax* hypnozoite (top) and schizont (bottom) at day 6 post-infection. (**B**) Immunofluorescent imaging of a 5hmC-negative *P. vivax* hypnozoite (top) and schizont (bottom) at day 7 post-infection. Yellow arrows indicate autofluorescence in the blue channel associated with cell debris above the hepatocyte monolayer. White arrows indicate hepatocyte nuclei which are dimly-stained with Hoechst 33342 and positive for 5mC or 5hmC. Purple arrows indicate 5mC-positive foci within the parasite. Bars represent 20 µm.

bioRxiv preprint doi: https://doi.org/10.1101/2023.01.31.526483; this version posted March 25, 2024. The copyright holder for this preprint (which was not certified by peer review) is the author/funder, who has granted bioRxiv a license to display the preprint in perpetuity. It is made available under aCC-BY 4.0 International license.

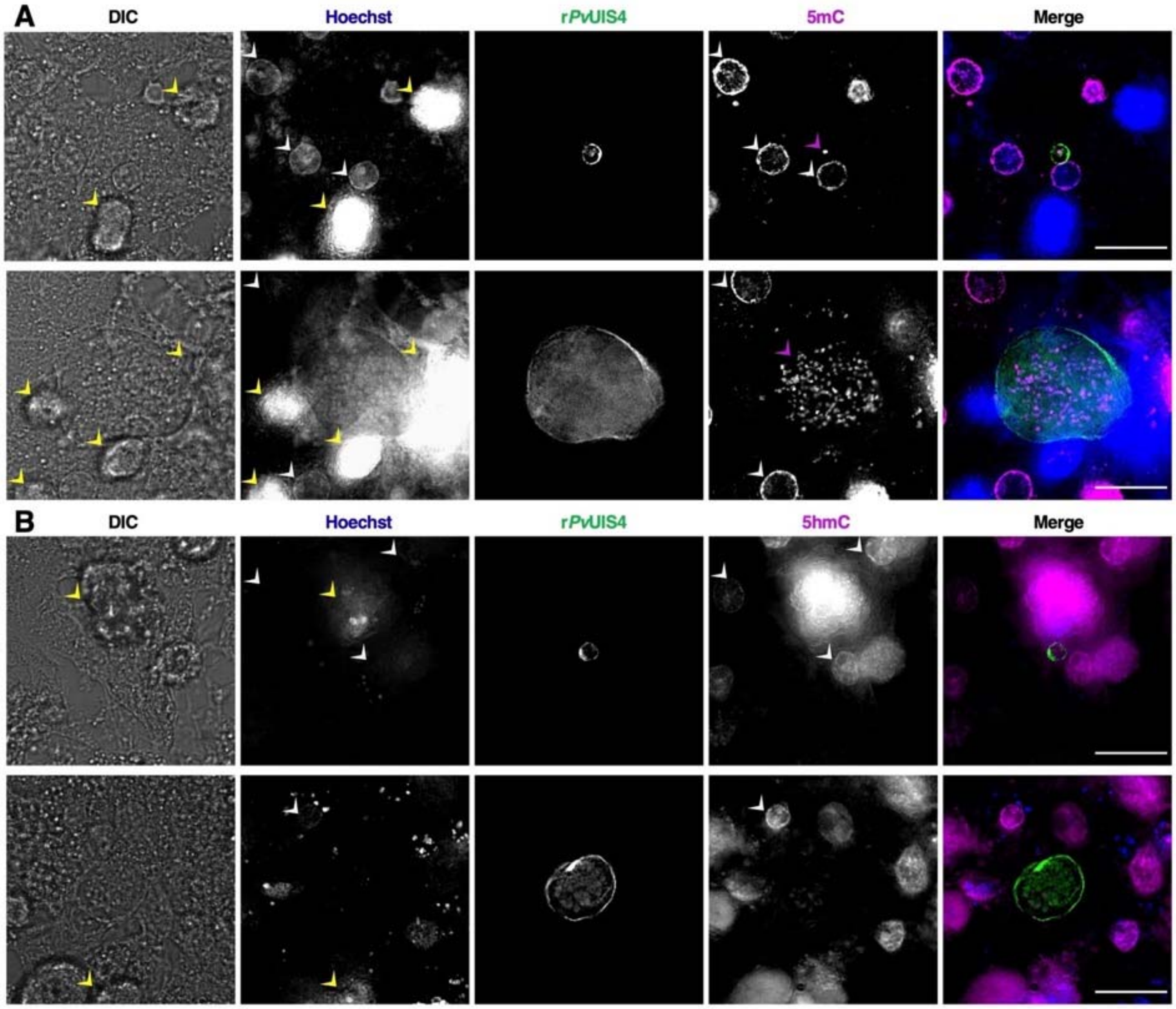

Fig. S7. Cytosine modifications in *P. vivax* liver forms, full panels from Case 2. (**A**) Immunofluorescent imaging of a 5mC-positive *P. vivax* hypnozoite (top) and schizont (bottom) at day 6 post-infection. (**B**) Immunofluorescent imaging of a 5hmC-negative *P. vivax* hypnozoite (top) and schizont (bottom) at day 7 post-infection. Yellow arrows indicate autofluorescence in the blue channel associated with cell debris above the hepatocyte monolayer. White arrows indicate hepatocyte nuclei which are dimly-stained with Hoechst 33342 and positive for 5mC or 5hmC. Purple arrows indicate 5mC-positive foci within the parasite. Bars represent 20 µm.

bioRxiv preprint doi: https://doi.org/10.1101/2023.01.31.526483; this version posted March 25, 2024. The copyright holder for this preprint (which was not certified by peer review) is the author/funder, who has granted bioRxiv a license to display the preprint in perpetuity. It is made available under aCC-BY 4.0 International license.

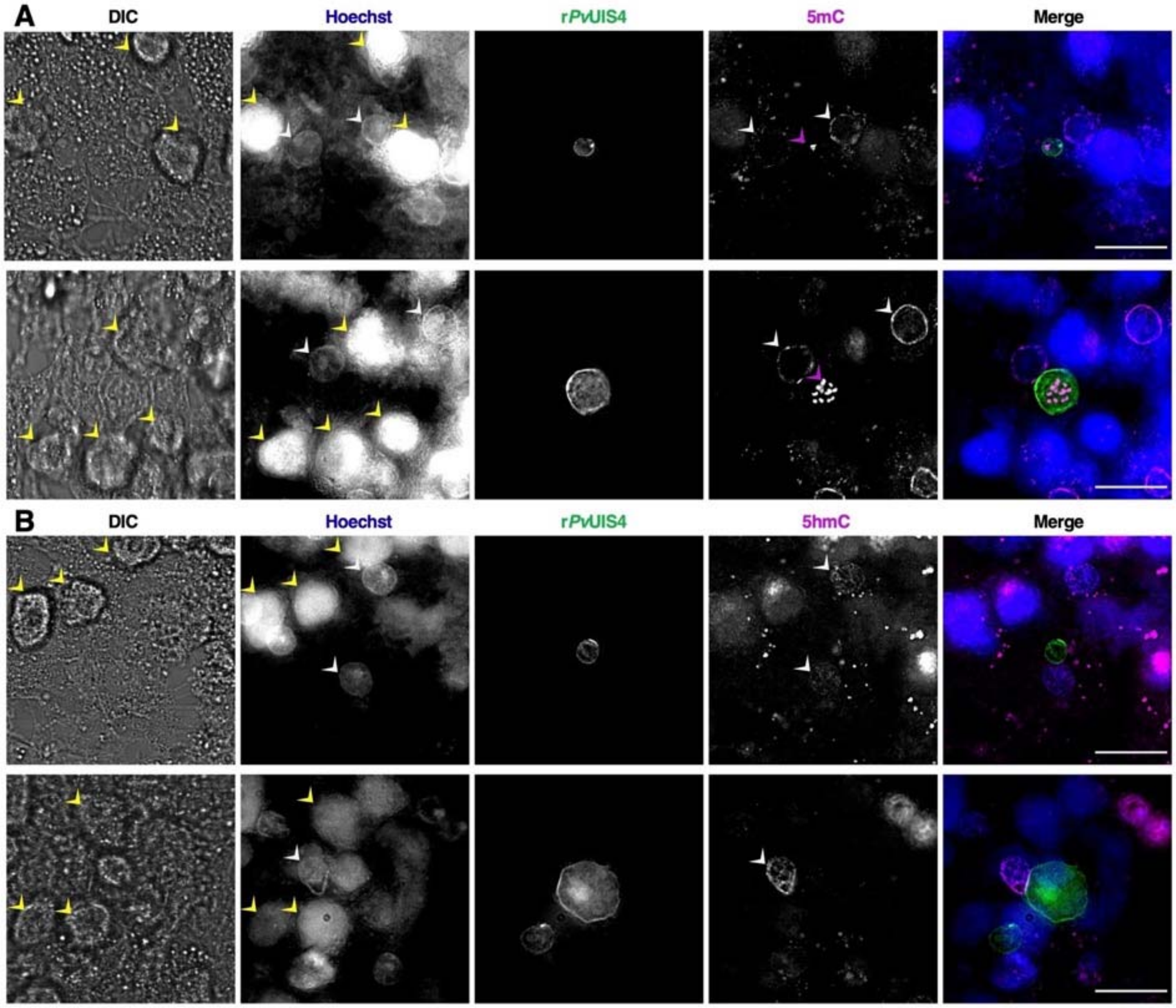

Fig. S8. Cytosine modifications in *P. vivax* liver forms, full panels from Case 3. (**A**) Immunofluorescent imaging of a 5mC-positive *P. vivax* hypnozoite (top) and schizont (bottom) at day 6 post-infection. (**B**) Immunofluorescent imaging of a 5hmC-negative *P. vivax* hypnozoite (top) and schizont (bottom) at day 7 post-infection. Yellow arrows indicate autofluorescence in the blue channel associated with cell debris above the hepatocyte monolayer. White arrows indicate hepatocyte nuclei which are dimly-stained with Hoechst 33342 and positive for 5mC or 5hmC. Purple arrows indicate 5mC-positive foci within the parasite. Bars represent 20 µm.

bioRxiv preprint doi: https://doi.org/10.1101/2023.01.31.526483; this version posted March 25, 2024. The copyright holder for this preprint (which was not certified by peer review) is the author/funder, who has granted bioRxiv a license to display the preprint in perpetuity. It is made available under aCC-BY 4.0 International license.

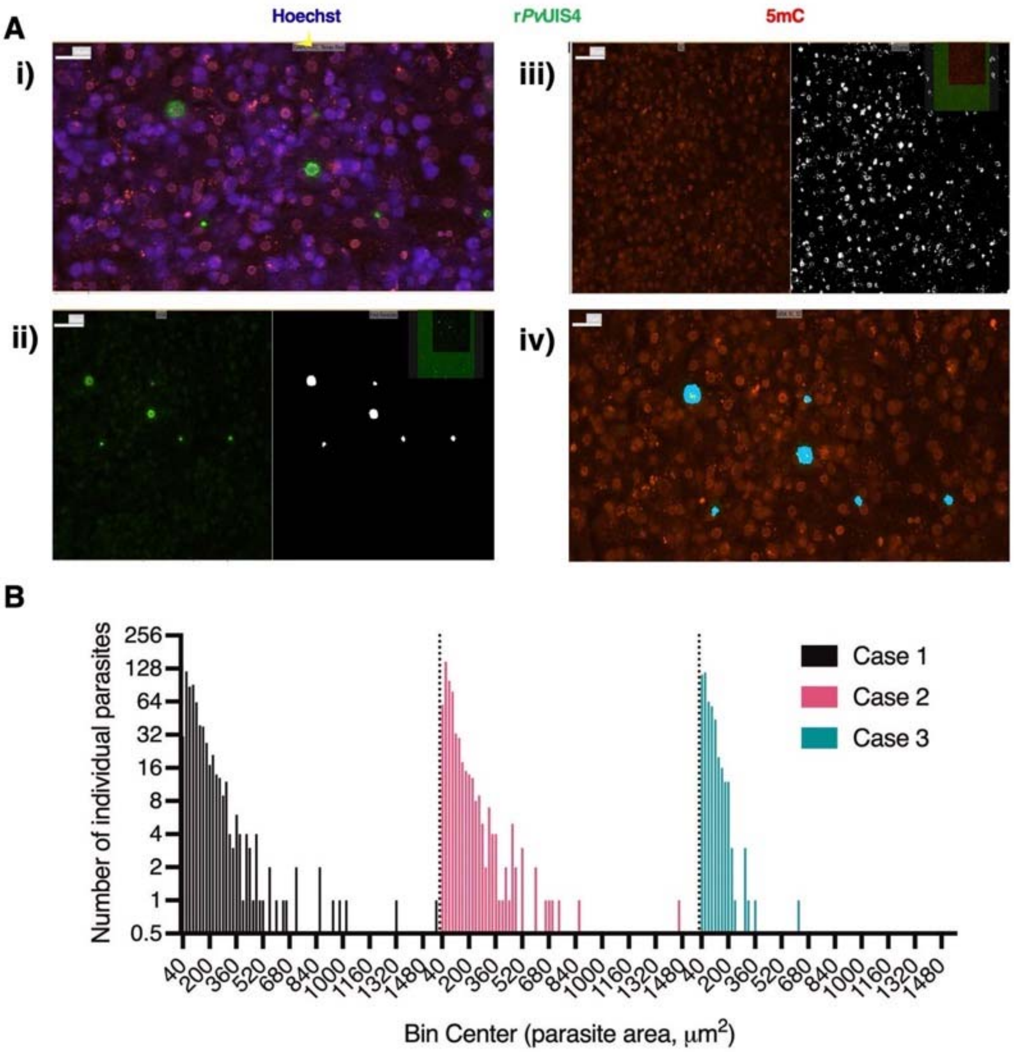

Fig. S9. High-content analysis of cytosine modifications and *P. vivax* liver stage population metrics. (**A**) Masks used to quantify parasite area and 5mC or 5hmC signal, i) raw image taken with a 20x objective, ii) mask for *P. vivax* liver stages, iii) mask for 5mC or 5hmC signal, iv) intersection of parasite mask (light blue) and 5mC or 5hmC signal mask (yellow), leading to quantified area of signal per form. **(B**) Histogram of growth area all parasites quantified for Case 1, 2, and 3 in Fig. 3. Hypnozoites were classified as forms with an area 125μm^2 and smaller.

bioRxiv preprint doi: https://doi.org/10.1101/2023.01.31.526483; this version posted March 25, 2024. The copyright holder for this preprint (which was not certified by peer review) is the author/funder, who has granted bioRxiv a license to display the preprint in perpetuity. It is made available under aCC-BY 4.0 International license.

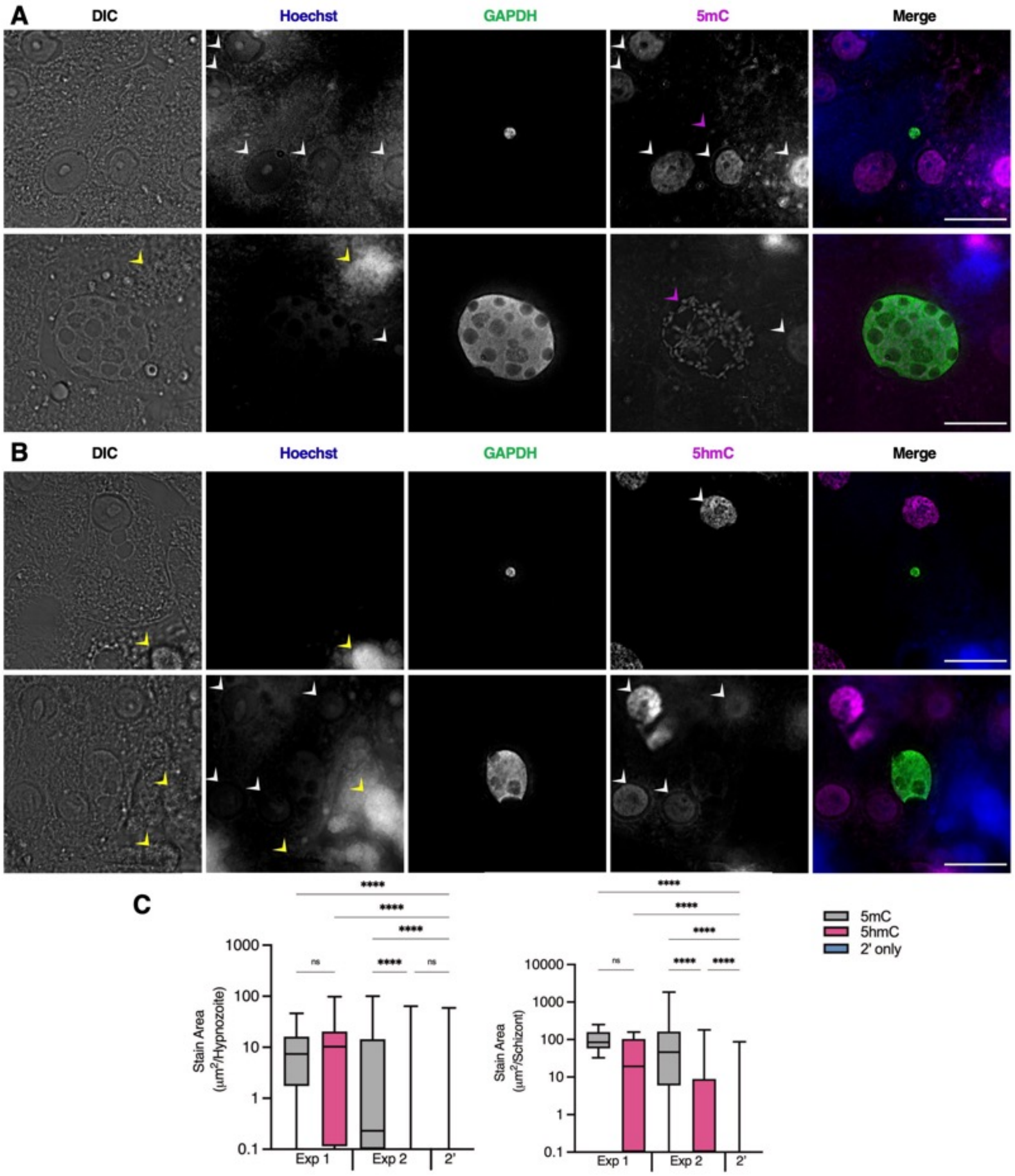

Fig. S10. Cytosine modifications in P. cynomolgi M/B strain liver forms. (**A**) Immunofluorescent imaging of a 5mC-positive P. cynomolgi hypnozoite (top) and schizont (bottom) at day 8 post-infection. (**B**) Immunofluorescent imaging of a 5hmC-negative P. cynomolgi hypnozoite (top) and schizont (bottom) at day 8 post-infection. Yellow arrows indicate autofluorescence in the blue channel associated with cell debris above the hepatocyte monolayer. White arrows indicate hepatocyte nuclei which are dimly-stained with Hoechst 33342 and positive for 5mC or 5hmC. Purple arrows indicate 5mC-positive foci within the parasite. Bars represent 20µm. (**C**) High-content quantification of 5mC or 5hmC stain area within hypnozoites or schizonts. Experiment 1 was fixed at day 8 post-infection, Experiment 2 was fixed at day 12 post-infection. Significance determined using Kruskal-Wallis tests for hypnozoites and schizonts, with Dunn's multiple comparisons, ****$p < .0001$, ns = not significant. Line, box and whiskers represent median, upper and lower quartiles, and minimum-to-maximum values, respectively, of all hypnozoites ($124 \leq n \leq 712$) or all schizonts ($7 \leq n \leq$

bioRxiv preprint doi: https://doi.org/10.1101/2023.01.31.526483; this version posted March 25, 2024. The copyright holder for this preprint (which was not certified by peer review) is the author/funder, who has granted bioRxiv a license to display the preprint in perpetuity. It is made available under aCC-BY 4.0 International license.

581) in culture, 2' indicates a secondary stain only control. Images in A and B are from Experiment 1.

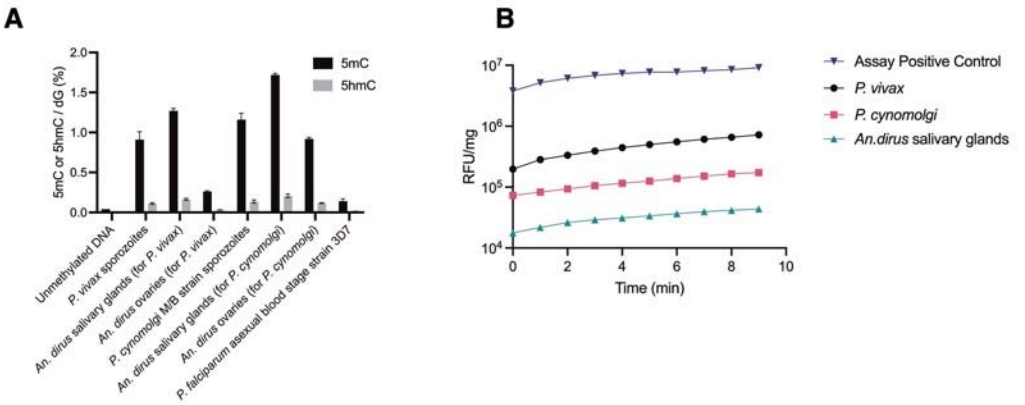

C

P. vivax	reads (#)	mapped reads (%)	mapped reads average genome coverage (x)	read methylation (%)	conversion rate (%)	average genome methylation (%)	percentage of cytosines meC/C >0.1 (%)
Sample 1	42,081,431	2.40	1.90	0.36	99.79	0.28	0.78
Sample 2	40,720,895	10.10	9.60	0.27	99.73	0.25	0.87
Sample 3	33,682,926	10.10	9.72	0.60	99.70	0.57	2.00
Sample 4	44,645,853	10.30	10.47	0.73	99.65	0.65	2.21
**average**		**8.23**		**0.49**	**99.72**		
**merged**	**161,141,105**		**31.69**			**0.49**	**0.89**
P. cynomolgi	**reads (#)**	**mapped reads (%)**	**mapped reads average genome coverage (x)**	**read methylation (%)**	**conversion rate (%)**	**average genome methylation (%)**	**percentage of cytosines meC/C >0.1 (%)**
Sample 1	52,657,816	1.40	2.08	0.48	99.73	0.28	1.00
Sample 2	36,325,051	0.60	0.52	0.88	99.78	0.49	1.06
Sample 3	39,109,832	1.80	4.00	0.39	99.75	0.27	1.06
Sample 4	18,476,083	7.70	2.18	0.70	99.78	0.62	1.82
**average**		**2.88**		**0.61**	**99.76**		
**merged**	**146,568,782**		**8.68**			**0.39**	**1.38**
P. falciparum	**reads (#)**	**mapped reads (%)**	**mapped reads average genome coverage (x)**	**read methylation (%)**	**conversion rate (%)**	**average genome methylation (%)**	**percentage of cytosines meC/C >0.1 (%)**
Sample 1	44,871,635	77.40	115.29	0.42	99.84	0.58	0.37

Fig. S11. Measurement of DNA methylation and DNA methyltransferase (DNMT) in *P. vivax* and *P. cynomolgi* sporozoites. (**A**) Liquid chromatography-tandem mass spectrometry (LC-MS/MS) analysis of 5mC or 5hmC from enzymatically-digested gDNA from *P. vivax* sporozoites, *P. cynomolgi* sporozoites, and *P. falciparum* blood stage parasites, as well as negative controls including uninfected mosquito salivary glands and ovaries from the same colony of mosquitoes used to generate the respective sporozoites. Bars represent SD of two independent experiments. (**B**) DNMT activity measured from nuclear extracts of *P. vivax* sporozoites, *P. cynomolgi* sporozoites, and uninfected mosquito salivary glands using the Epiquick DNMT activity assay. Data are from a single experiment. (**C**) Summary statistics of read sets, percentage of mapped reads, read methylation levels, conversion rate and genome-wide methylation levels from bisulfite sequencing.

bioRxiv preprint doi: https://doi.org/10.1101/2023.01.31.526483; this version posted March 25, 2024. The copyright holder for this preprint (which was not certified by peer review) is the author/funder, who has granted bioRxiv a license to display the preprint in perpetuity. It is made available under aCC-BY 4.0 International license.

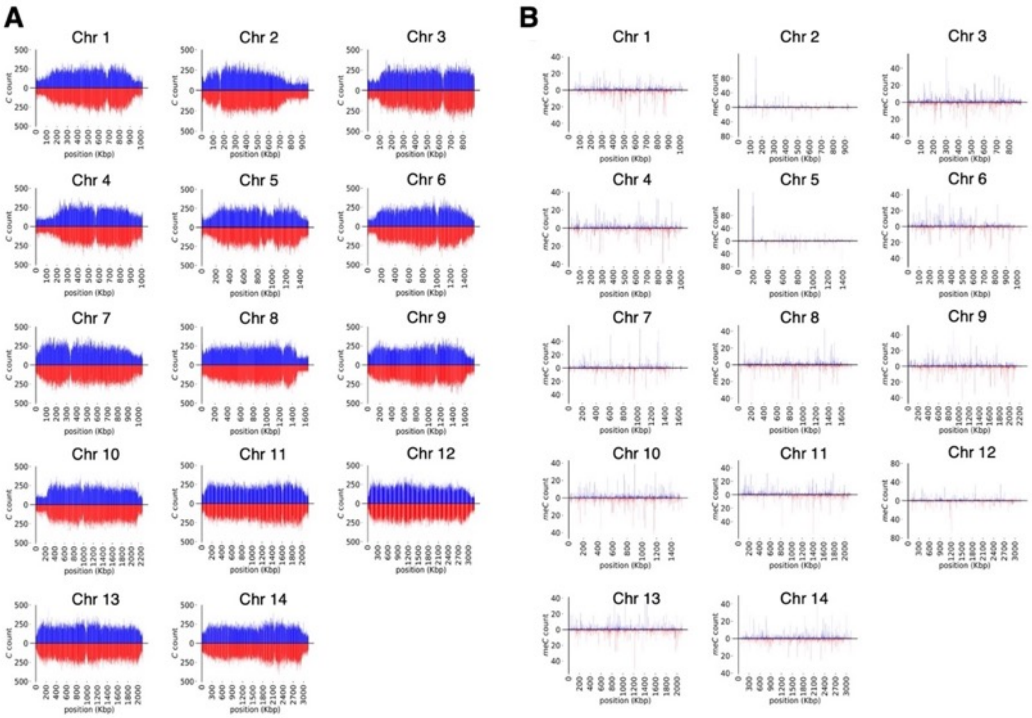

Fig. S12. Cytosine and methylation density plots for *P. vivax* sporozoites. (**A**) CG content of chromosome 1 to 14 (Chr 1-14). The total number of cytosines quantified on each strand using 1 kbp long non-overlapping windows. (**B**) The total number of methylated cytosines quantified on each strand using 1 kbp long non-overlapping windows.

bioRxiv preprint doi: https://doi.org/10.1101/2023.01.31.526483; this version posted March 25, 2024. The copyright holder for this preprint (which was not certified by peer review) is the author/funder, who has granted bioRxiv a license to display the preprint in perpetuity. It is made available under aCC-BY 4.0 International license.

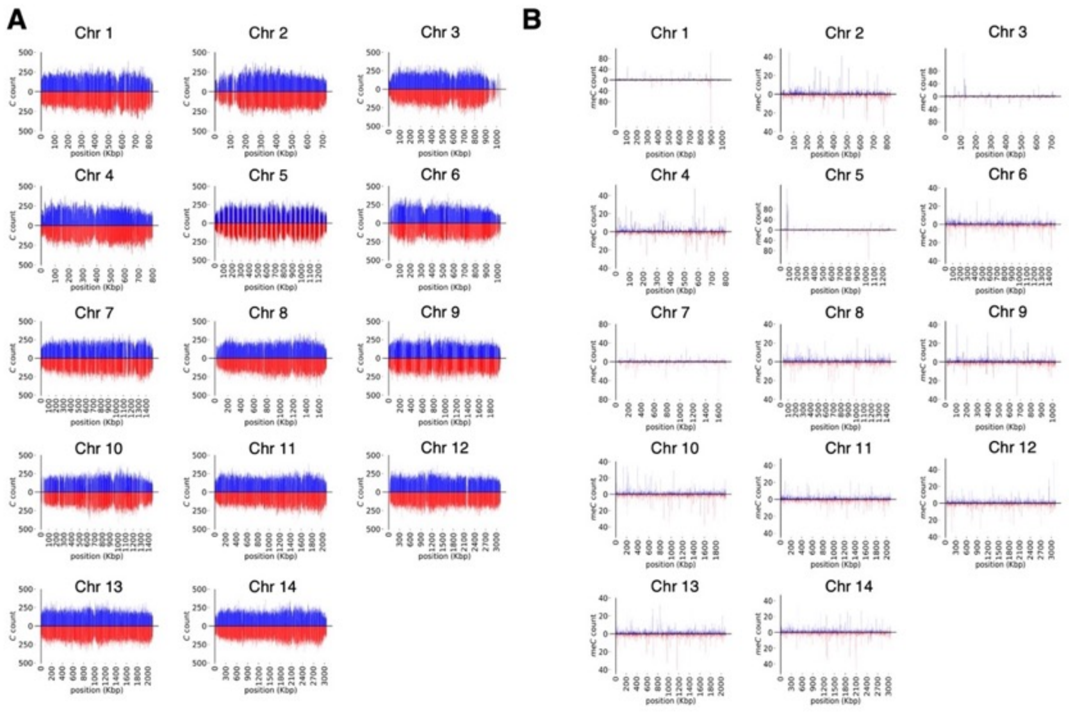

Fig. S13. Cytosine and methylation density plots for *P. cynomolgi* sporozoites. (**A**) CG content of chromosome 1 to 14 (Chr 1-14). The total number of cytosines quantified on each strand using 1 kbp long non-overlapping windows. (**B**) The total number of methylated cytosines quantified on each strand using 1 kbp long non-overlapping windows.

bioRxiv preprint doi: https://doi.org/10.1101/2023.01.31.526483; this version posted March 25, 2024. The copyright holder for this preprint (which was not certified by peer review) is the author/funder, who has granted bioRxiv a license to display the preprint in perpetuity. It is made available under aCC-BY 4.0 International license.

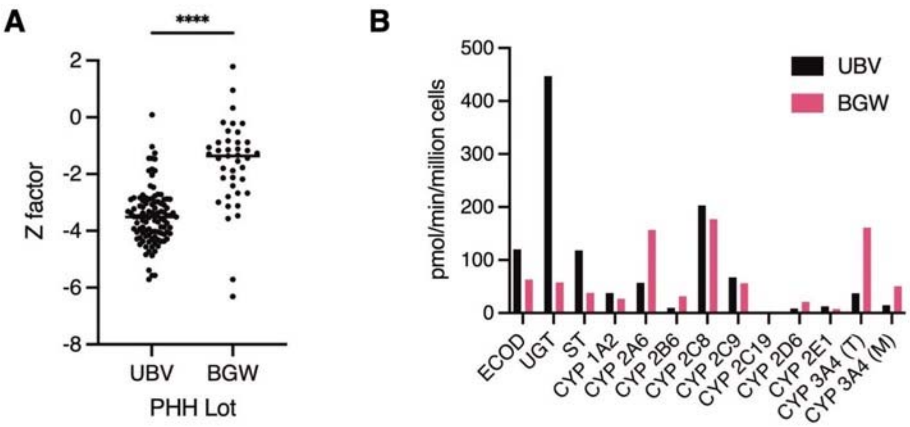

Fig. S14. Monensin activity in all control wells based on PHH lot. (**A**) Initially, the ReFRAME was screened with cryopreserved vials of a specific lot of PHH, UBV. Screening continued with a new lot, BGW, once the supply of UBV vials was exhausted. The activity of monensin was significantly reduced in wells with BGW versus UBV PHH. A Mann-Whitney test indicates the difference was statistically significant, $U(N_{\mathrm{UBV}}=105, N_{\mathrm{BGW}}=41) = 552$, $z = -2.15$, ****$p < .0001$. (**B**) Metabolic activity panel for PHH lots UBV and BGW performed as part of regular quality-control at the vendor (BioIVT). ECOD: 7-ethoxycoumarin O-deethylation, UGT: 7-hydroxycoumarin glucuronidation, ST: 7-hydroxycoumarin sulfation, CYP 1A2: phenacetin O-deethylation, CYP 2A6: coumarin 7-hydroxylation, CYP 2B6: bupropion hydroxylation, CYP 2C8: amodiaquine N-desethylation, CYP 2C9: tolbutamide methyl-hydroxylation, CYP 2C19: S-mephenytoin 4'-hydroxylation, CYP 2D6: dextromethorphan O-demethylation, CYP 2E1: chlorzoxazone 6-hydroxylation, CYP 3A4 (T): testosterone 6β-hydroxylation, CYP 3A4 (M): midazolam 1-hydroxylation.

bioRxiv preprint doi: https://doi.org/10.1101/2023.01.31.526483; this version posted March 25, 2024. The copyright holder for this preprint (which was not certified by peer review) is the author/funder, who has granted bioRxiv a license to display the preprint in perpetuity. It is made available under aCC-BY 4.0 International license.

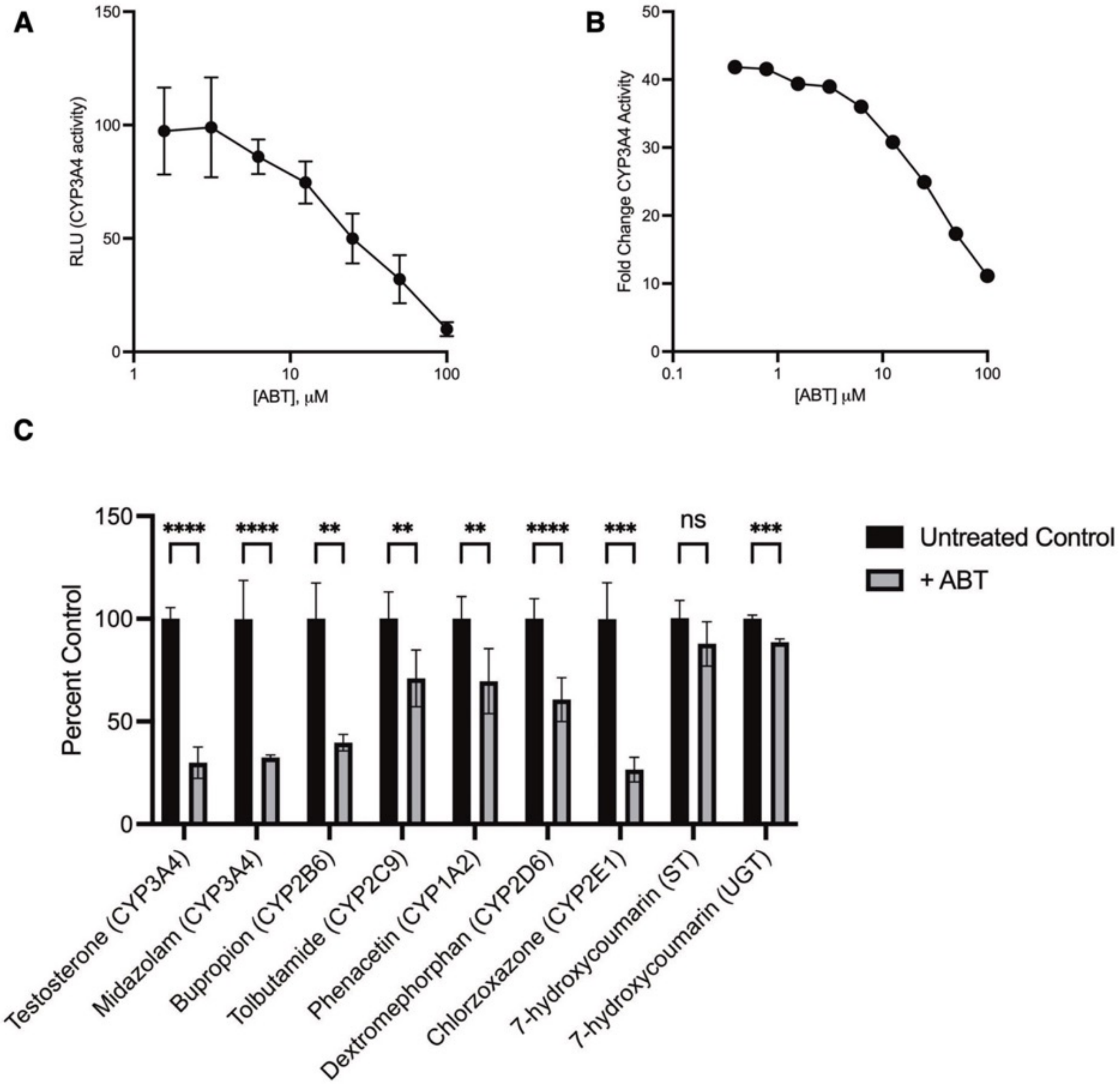

Fig. S15. Characterization of primary human hepatocyte (PHH) metabolism following 1-aminobenzotriazole (1-ABT) treatment. (**A**) PHH lot BGW were seeded into 384-well plates and cultured for 7 days before addition of a dilution series of 1-ABT in media. Cytochrome P450 3A4 activity (CYP3A4) was measured using luciferin-IPA (Promega). RLU: relative luminescence units. Bars represent SD of quadruplicate wells. Data are representative of two independent experiments. (**B**) PHH lot BGW were cultured in 384-well plates before addition of 25 μM rifampicin in media on days 4 and 6 to induce CYP3A4 expression. At day 7 post-seed, CYP3A4 activity was measured by adding luciferin-IPA and a dilution series of 1-ABT in media. Fold change was calculated based on matching uninduced controls. Data are from one independent experiment. (**C**) PHH lot BGW were seeded in 384-well plates and cultured for 7 days before treatment with 100 μM 1-ABT for 1h, followed by addition of substrates for 1 h and collection for analysis by mass spectrometry. Data are combined from two independent experiments, bars represent SD of all replicates. Significance determined by student's t tests, ****p <.0001, ***p <.001, **p <.01, ns, not significant.

bioRxiv preprint doi: https://doi.org/10.1101/2023.01.31.526483; this version posted March 25, 2024. The copyright holder for this preprint (which was not certified by peer review) is the author/funder, who has granted bioRxiv a license to display the preprint in perpetuity. It is made available under aCC-BY 4.0 International license.

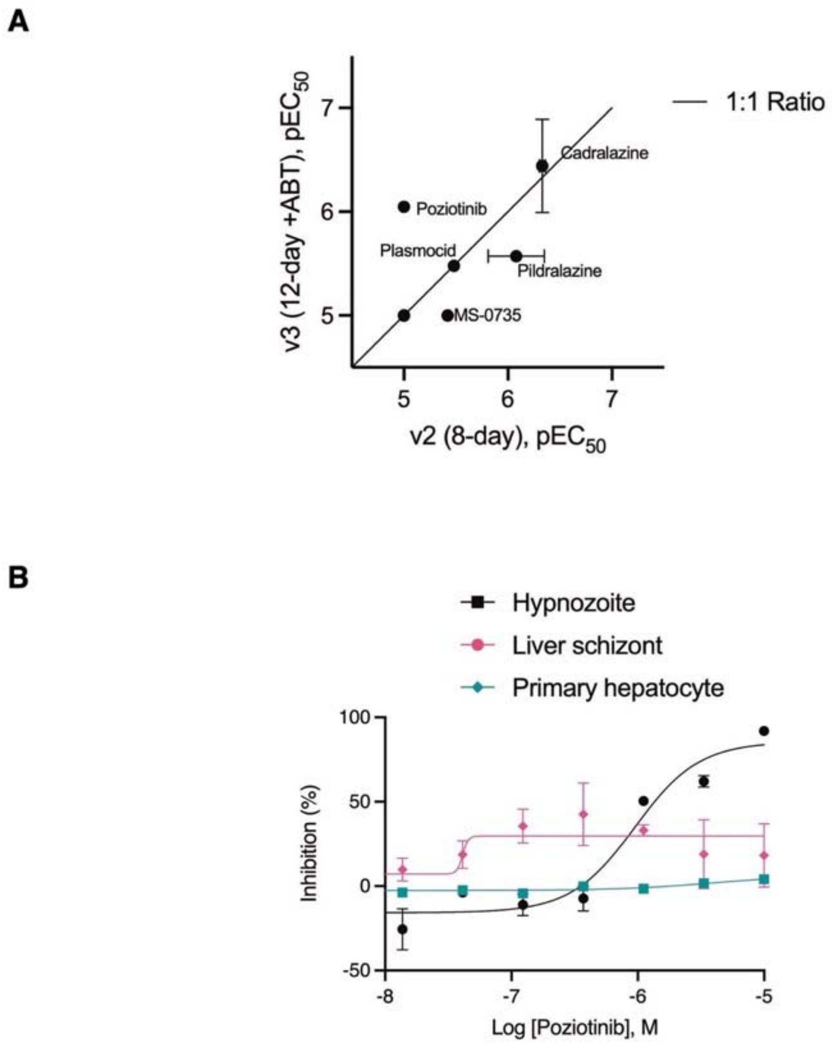

Fig. S16. ReFRAME hits re-confirmed in a *P. vivax* 12-day 1-ABT assay. (**A**) Hypnozonticidal potency comparison of 12 ReFRAME hits in 8-day and 12-day 1-ABT dose-response confirmation assays. Cadralazine, plasmocid, and pidralazine potencies were unaffected by assay version, while MS-0735 was less potent, and poziotinib was more potent, in the 12-day 1-ABT assay. Budralazine, dramedilol, RGH-5526, dihydralazine, todralazine, endralazine, and mopidralazine were inactive (pEC50 < 5) regardless of assay version. (**B**) Dose-response chart of poziotinib activity in the 12-day 1-ABT assay, pEC_{50} against hypnozoites = 6.05. Bars represent SEM.

bioRxiv preprint doi: https://doi.org/10.1101/2023.01.31.526483; this version posted March 25, 2024. The copyright holder for this preprint (which was not certified by peer review) is the author/funder, who has granted bioRxiv a license to display the preprint in perpetuity. It is made available under aCC-BY 4.0 International license.

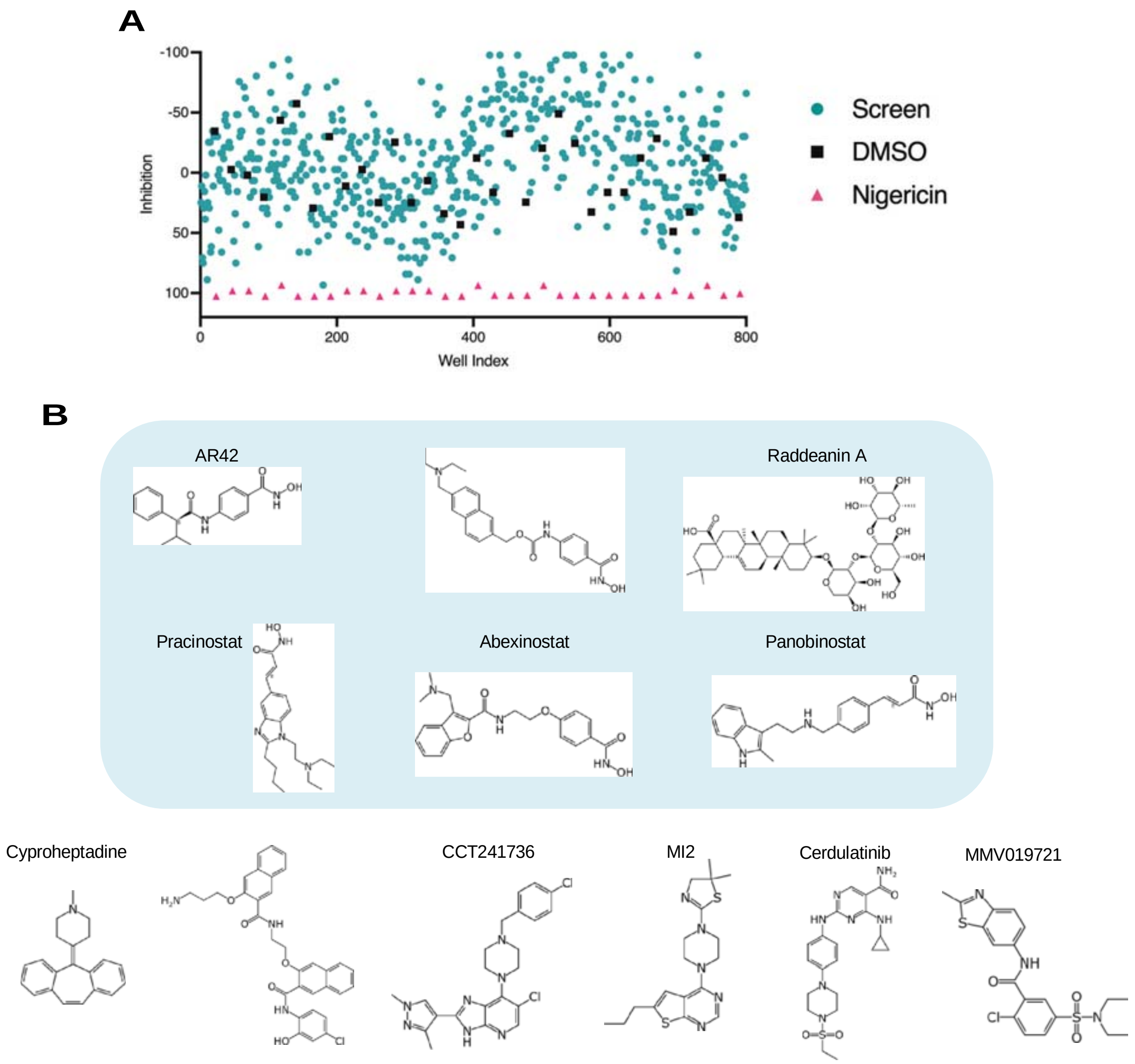

Fig. S17. Epigenetic Inhibitor library screen and hits. (**A**) Index chart of an epigenetic inhibitor library screened against *P. vivax* hypnozoites in a v3 (12-day 1-ABT) assay. Teal circle: library, black square: DMSO, pink triangle: 200 nM nigericin. (**B**) Structures of epigenetic inhibitor hits which were confirmed to be active against *P. vivax* hypnozoites in dose-response assays; blue: histone deacetylase inhibitors.

bioRxiv preprint doi: https://doi.org/10.1101/2023.01.31.526483; this version posted March 25, 2024. The copyright holder for this preprint (which was not certified by peer review) is the author/funder, who has granted bioRxiv a license to display the preprint in perpetuity. It is made available under aCC-BY 4.0 International license.

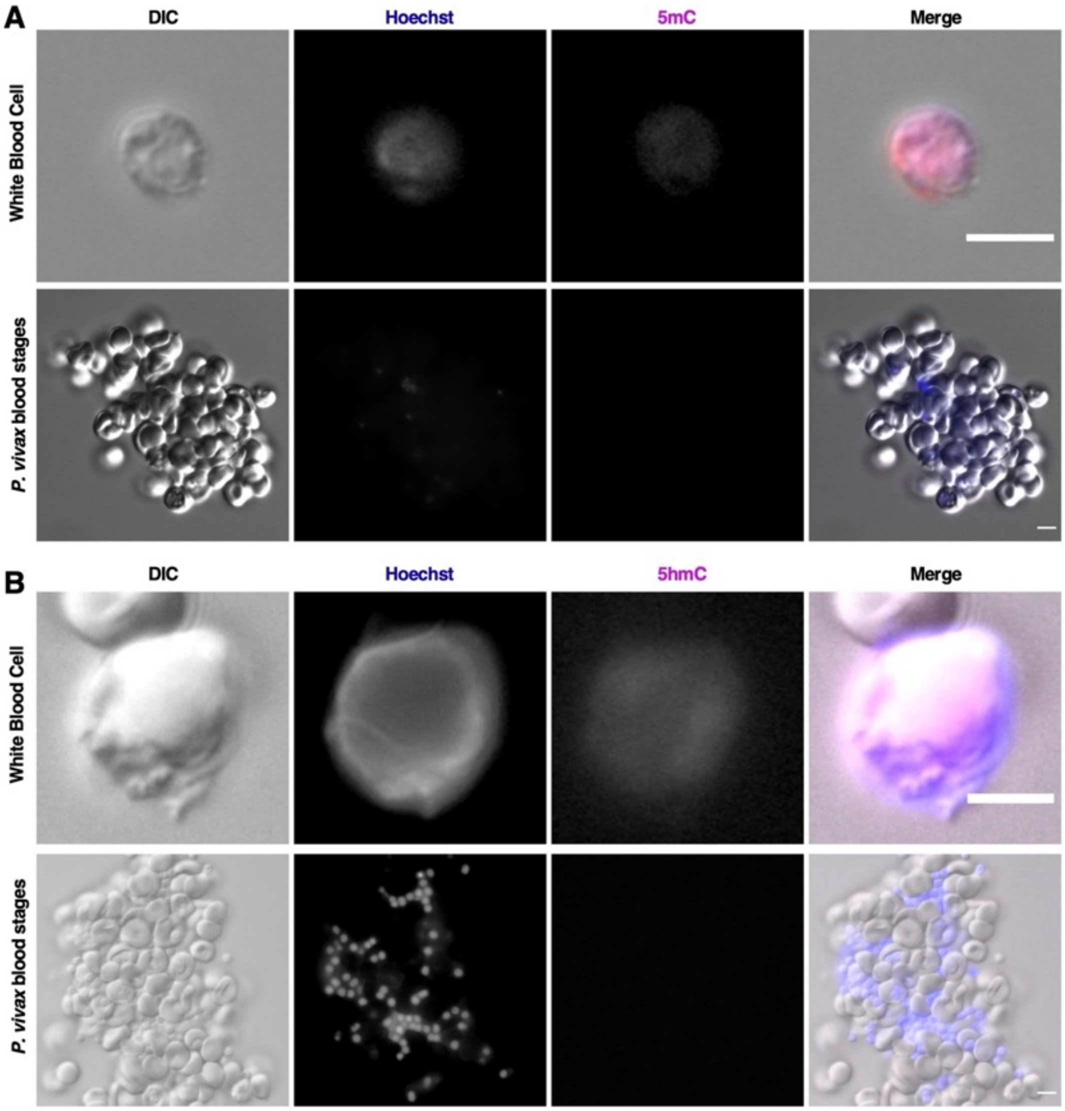

Fig. S18. Cytosine modification in *P. vivax* blood stages. (**A**) *P. vivax* blood stages from patient isolates appeared negative when stained with 5mC. A white blood cell positive for 5mC serves as a stain control. (**B**) *P. vivax* blood stages from patient isolates appeared negative when stained with 5hmC. A white blood cell positive for 5hmC serves as a stain control. Bars represent 10 µm.

bioRxiv preprint doi: https://doi.org/10.1101/2023.01.31.526483; this version posted March 25, 2024. The copyright holder for this preprint (which was not certified by peer review) is the author/funder, who has granted bioRxiv a license to display the preprint in perpetuity. It is made available under aCC-BY 4.0 International license.

Data S1. (separate file)
Details for chemistry (batches, suppliers, structures); individual run data used to calculate all pEC_{50}s and pCC_{50}s; details for biologicals; details for reagents and replication.

Data S2. (separate file)
Raw pharmacokinetic data report from Wuxi for cadralazine in rhesus macaques.

Data S3. (separate file)
Contents of the Targetmol Epigenetic Library
